# Magneto‐NIR‐II‐Programmed Cascade Nanozymes Unlocking Blood–Brain Barrier Translocation and Autophagic Resistance in Glioblastoma

**DOI:** 10.1002/advs.76998

**Published:** 2026-08-05

**Authors:** Ruocan Liu, Yundi Wu, Shuai Zhang, Hongjuan Zhao, Jiping Zheng, Shiyang Shao, Jianqiang Chen, Xilong Wu

**Affiliations:** ^1^ State Key Laboratory of Digital Medical Engineering School of Biomedical Engineering School of Pharmaceutical Sciences Hainan University Haikou China; ^2^ Key Laboratory of Tropical Biological Resources of Ministry of Education Hainan Engineering Research Center for Drug Screening and Evaluation Collaborative Innovation Center of One Health Hainan University Haikou China; ^3^ Key Laboratory of Emergency and Trauma of Ministry of Education Engineering Research Center for Hainan Biological Sample Resources of Major Diseases The First Affiliated Hospital Hainan Medical University Haikou China; ^4^ School of Life and Health Sciences Collaborative Innovation Center of Life and Health Hainan University Haikou China; ^5^ School of Materials Science and Engineering Hainan University Haikou China

**Keywords:** autophagy suppression, blood–brain barrier, cascade nanozymes, glioblastoma, magnetic guidance, NIR‐II photodynamic therapy

## Abstract

Glioblastoma (GBM) remains a highly aggressive central nervous system malignancy, and its treatment is hindered by poor drug accumulation across the blood–brain barrier (BBB) and autophagy‐mediated repair. To address these barriers, rare‐earth‐doped Nd_0.02_Fe_2.98_S_4_@HA nanozymes (NFSH) are constructed as magneto‐NIR‐II‐programmed cascade nanozymes for trans‐BBB delivery, multimodal imaging, and ferroptosis amplification. Hyaluronic acid (HA)‐mediated CD44 targeting and oriented magnetic field‐enhanced BBB permeability promote tumor enrichment, while Nd^3+^ doping endows NFSH with strengthened superparamagnetism, near‐infrared second window (NIR‐II) photodynamic activity, and NIR‐II fluorescence capability. Under alternating magnetic field (AMF) and NIR‐II laser stimulation, NFSH activates catalase‐, peroxidase‐, glutathione oxidase‐, and nicotinamide adenine dinucleotide (NADH) oxidase‐like cascade catalysis, which amplifies reactive oxygen species (ROS) production, consumes glutathione, and induces ferroptosis. In the acidic tumor microenvironment, AMF further promotes H_2_S release, disrupts lysosomal autophagic degradation, and aggravates mitophagy inhibition through NADH depletion‐mediated ATP deficiency. This cascade mechanism enhances ferroptosis and reshapes the tumor immune microenvironment by relieving hypoxia and promoting M2‐to‐M1 macrophage polarization. In addition, NFSH enables NIR‐II fluorescence and T_2_‐weighted magnetic resonance imaging for real‐time visualization of treatment. This strategy provides an integrated trans‐BBB theranostic platform for autophagy‐suppressed ferroptosis therapy against GBM.

## Graphical abstract

Magneto‐NIR‐II‐programmed NFSH nanozymes integrate magnetic blood–brain barrier (BBB) translocation, CD44 targeting, multimodal imaging, and cascade catalytic therapy for glioblastoma. Alternating magnetic fields and NIR‐II irradiation amplify ferroptosis, release H_2_S, suppress autophagic and mitophagic repair, and reshape the immune microenvironment, offering a trans‐BBB theranostic strategy for resistant brain tumors.

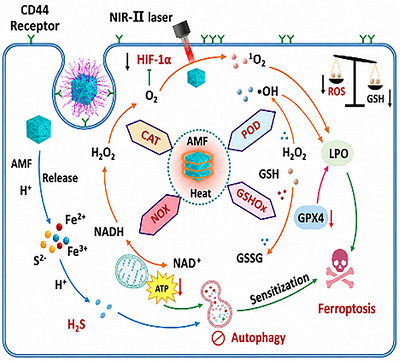

## 1 Introduction

Glioblastoma (GBM) is the most aggressive primary tumor of the central nervous system and accounts for approximately 50% of malignant neural tumors [1]. Its treatment remains difficult because the blood–brain barrier (BBB) restricts therapeutic entry into the brain, while the highly invasive growth pattern of GBM reduces local drug retention in tumor tissue. Therefore, an effective GBM therapy must not only cross the BBB but also achieve tumor‐selective accumulation and real‐time therapeutic visualization. Nanotechnology offers multiple strategies for improving BBB penetration and GBM diagnosis and treatment [2]. External fields, including focused ultrasound [3, 4], microwaves [5], and lasers [6, 7], can facilitate nanosystem transport across the BBB. However, these approaches may compromise BBB integrity and increase the risk of nonspecific entry of harmful substances into the brain. In contrast, magnetic field‐mediated delivery provides a more controllable strategy because it can guide magnetic nanoparticles toward deep brain lesions while helping maintain BBB structure. Magnetic nanoparticles have been used for oriented magnetic field (OMF)‐guided targeting of deep intracranial lesions [8, 9, 10], suggesting their potential for GBM therapy. Beyond delivery, magnetic nanoparticles can also integrate diagnosis and therapy when their magnetic properties are coupled with optical functions. In this context, rare‐earth ion doping provides an effective route to regulate the electronic and magnetic structures of iron sulfide‐based nanomaterials. In particular, neodymium (Nd) doping can strengthen superparamagnetism and introduce near‐infrared second‐window (NIR‐II) fluorescence and photodynamic capabilities, thereby supporting OMF‐assisted BBB crossing, NIR‐II imaging, and NIR‐II‐triggered therapy within a single nanoplatform. Such a design is highly desirable for GBM because it connects trans‐BBB delivery, tumor enrichment, and image‐guided treatment in one therapeutic sequence.

Nanozymes, as artificial enzyme‐like nanomaterials, attract increasing attention in biomedicine because they provide stable, tunable, and multifunctional catalytic activities [11, 12, 13]. In antitumor therapy, many nanozymes generate cytotoxic hydroxyl radicals (•OH) through Fenton‐like reactions and thereby induce oxidative damage [14]. However, the tumor microenvironment (TME) limits this therapeutic effect through mild acidity, hypoxia, and high glutathione (GSH) levels, which reduce ROS accumulation and protect tumor cells from oxidative death. Metal ion doping and crystal structure modulation improve nanozyme catalysis [15, 16], but catalytic efficiency still requires further enhancement in complex tumors. Heat can accelerate enzyme activity [17, 18], and alternating magnetic field (AMF)‐induced magnetothermal activation offers deeper penetration than photothermal heating. Our previous studies also demonstrate that magnetic heating enhances nanozyme catalysis under weakly acidic and GSH‐rich conditions [19]. In parallel, integrating nanozymes with photodynamic therapy (PDT) provides another route to reinforce oxidative stress [20]. Near‐infrared second window (NIR‐II, 1000–1800 nm) PDT offers deeper tissue penetration and lower light scattering than conventional optical windows [21, 22, 23], but nanozymes that combine NIR‐II activation, magnetic response, and cascade catalysis remain limited.

Ferroptosis provides a promising direction for nanozyme‐based cancer therapy because this regulated cell death depends on iron‐associated lipid peroxidation and intracellular ROS accumulation [24]. However, tumor cells can activate autophagy‐related repair pathways, especially mitophagy, to remove damaged mitochondria and resist ferroptotic stress. Therefore, blocking protective autophagic degradation may sensitize tumor cells to ferroptosis. In this context, nicotinamide adenine dinucleotide (NADH) oxidase (NOX)‐like activity deserves attention because natural NOX enzymes transfer electrons from intracellular reducing equivalents to oxygen and generate ROS, thereby linking redox imbalance with mitochondrial dysfunction [25, 26]. For nanozymes, NOX‐like catalysis can serve as an upstream oxidative trigger that aggravates mitochondrial damage, disturbs cellular energy metabolism, and increases vulnerability to lipid peroxidation [27]. Hydrogen sulfide (H_2_S), an endogenous signaling molecule, also regulates autophagy, metabolism, and immunity [28, 29, 30]. H_2_S‐based therapeutic strategies can modulate redox enzyme activity and induce tumor cell death [31, 32], and emerging evidence links H_2_S to mitophagy regulation [33, 34]. However, the mechanism by which H_2_S blocks mitophagic degradation and amplifies ferroptosis remains insufficiently defined. More importantly, the cooperative inhibition of autophagic repair by H_2_S‐induced lysosomal dysfunction and NOX‐like activity‐driven mitochondrial damage has rarely been explored in tumor therapy. This unresolved mechanism provides a key rationale for designing cascade nanozymes that simultaneously amplify ROS stress, consume reducing substrates, disrupt mitophagy, and enhance ferroptosis in GBM.

**SCHEME 1:**
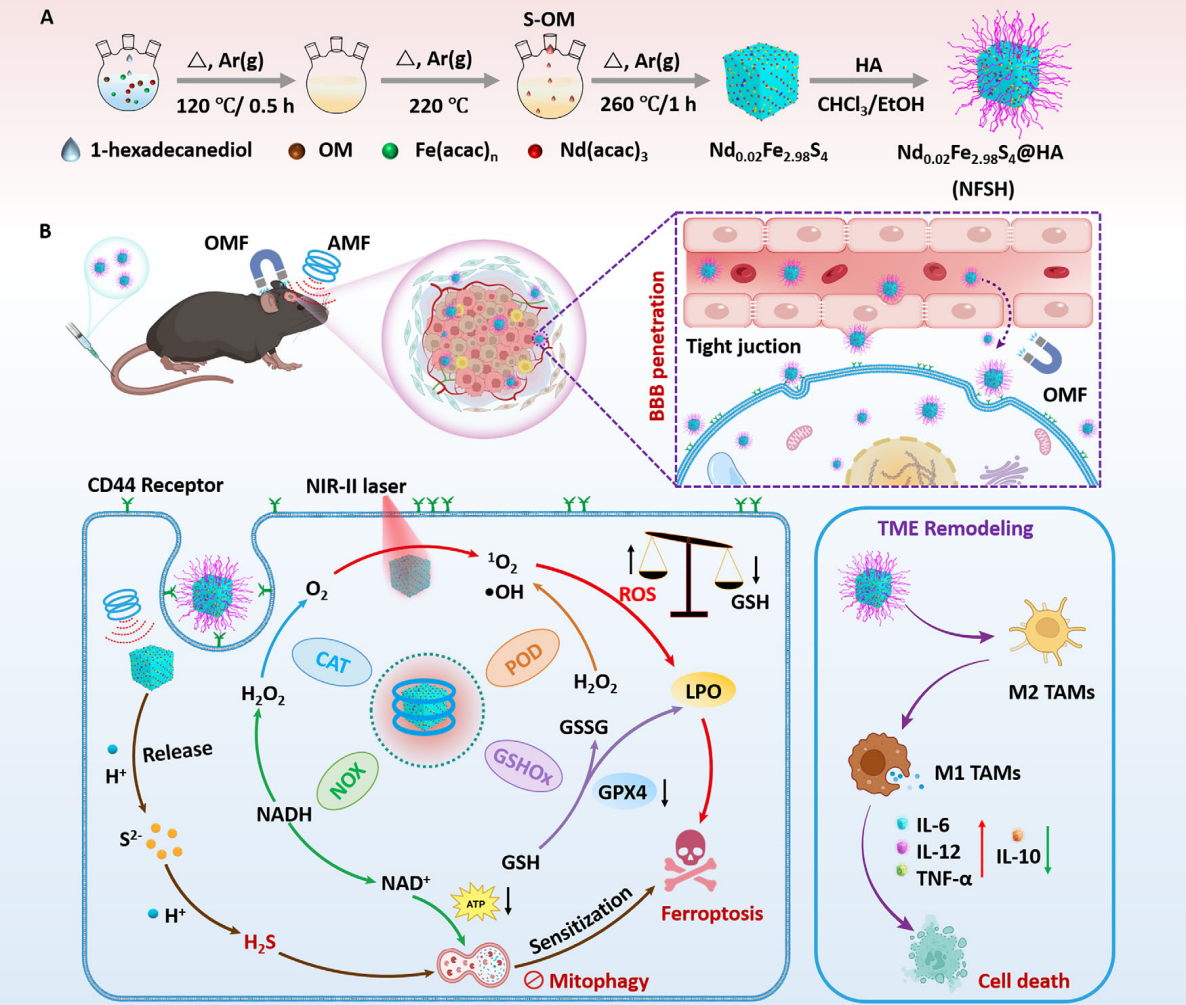
Synthesis and therapeutic workflow of NFSH nanozymes. (A) The preparation process for NFSH nanozymes. (B) Schematic illustration of the in vivo treatment procedure after intravenous administration of NFSH nanozymes, including OMF‐assisted BBB permeation, HA‐mediated CD44 targeting, tumor‐cell internalization, AMF‐ and 1064 nm laser‐triggered ROS generation and GSH consumption, acidic microenvironment‐ and AMF‐responsive H_2_S release, NADH depletion, mitophagy inhibition, ferroptosis induction, and TIME remodeling.

Herein, a Nd^3+^‐doped non‐stoichiometric pyrrhotite compound (Nd_0.02_F_2.98_S_4_@HA), termed NFSH, is constructed as magneto‐NIR‐II‐programmed cascade nanozymes for trans‐BBB delivery, multimodal imaging, and ferroptosis‐amplified GBM therapy (Scheme 1). Hyaluronic acid (HA) modification enables CD44‐mediated tumor targeting, while OMF enhances BBB permeability and promotes intracranial tumor enrichment. Nd^3+^ doping strengthens the magnetic response of NFSH and endows the nanozyme with NIR‐II fluorescence and NIR‐II PDT capability. Under AMF and NIR‐II laser stimulation, NFSH activates catalase (CAT)‐, peroxidase (POD)‐, glutathione oxidase (GSHOx)‐, and NOX‐like catalytic functions. This multienzyme cascade increases ROS generation, accelerates GSH depletion, relieves hypoxia, and promotes lipid peroxidation, thereby inducing ferroptosis. In the acidic TME, AMF further promotes H_2_S release from NFSH. Released H_2_S impairs lysosomal function and blocks autophagic degradation, while NOX‐like activity‐associated NADH depletion disrupts adenosine triphosphate (ATP) generation and aggravates mitophagy inhibition. This coordinated mechanism suppresses autophagic repair and sensitizes GBM cells to ferroptosis. Meanwhile, AMF/NIR‐II dual stimulation reshapes the tumor immune microenvironment (TIME) by alleviating hypoxia and promoting macrophage repolarization from the M2 phenotype toward the M1 phenotype. NFSH also enables NIR‐II fluorescence imaging and T_2_‐weighted (T_2_w) magnetic resonance imaging (MRI), supporting real‐time visualization of tumor accumulation and therapeutic progression. Overall, this work establishes a magnetically guided and NIR‐II‐programmed nanozyme strategy that addresses both trans‐BBB delivery and autophagic resistance, offering an integrated theranostic approach for ferroptosis‐based GBM treatment.

## 2 Results and Discussion

### 2.1 Structural and Physicochemical Characterization of NFSH Nanozymes

**FIGURE 1:**
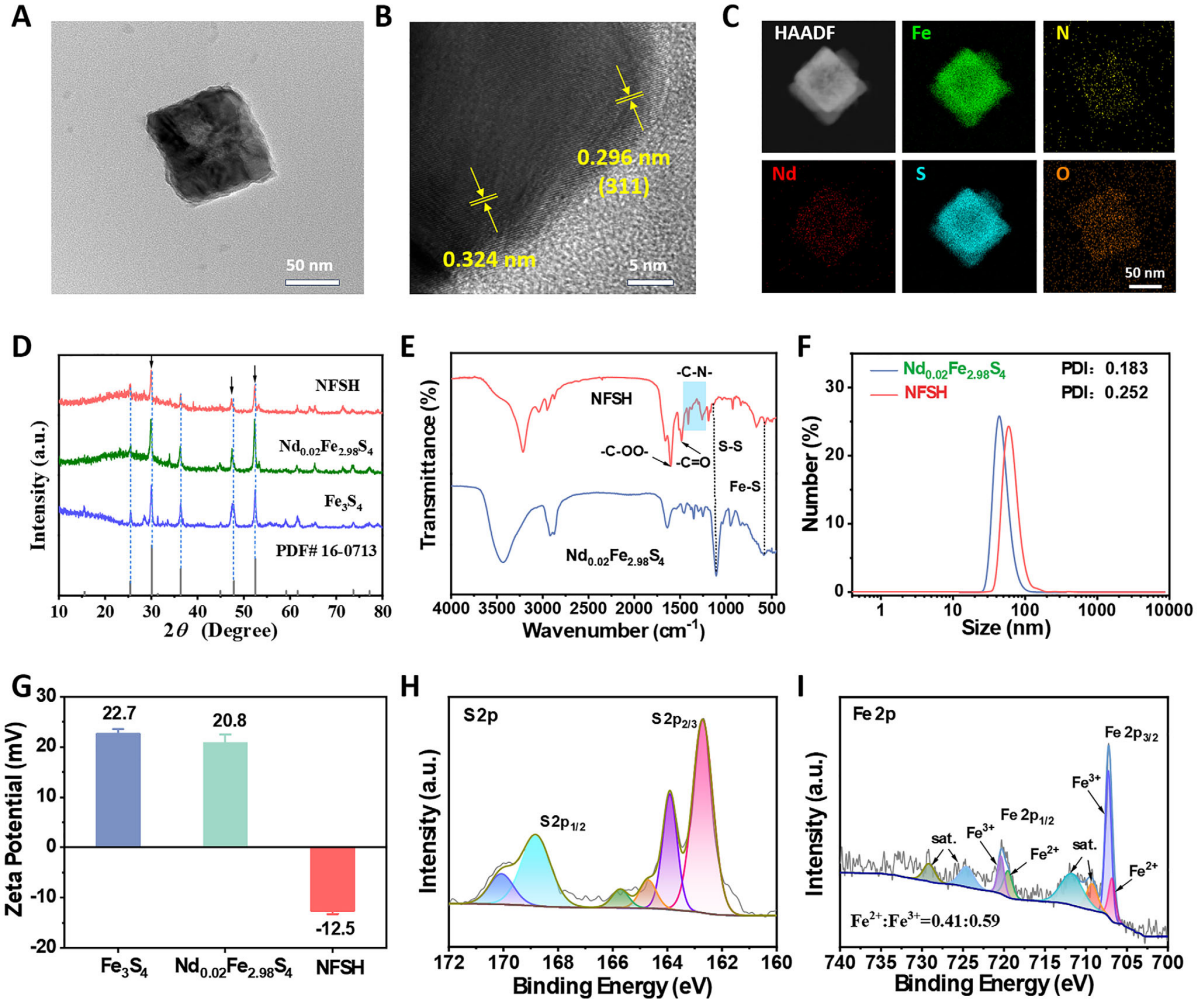
Structural and physicochemical characterization of NFSH nanozymes. (A) TEM image and (B) HRTEM image of Nd_0.02_Fe_2.98_S_4_ nanoparticles. (C) Elemental mapping images of Nd_0.02_Fe_2.98_S_4_ nanoparticles. (D) XRD patterns of Fe_3_S_4_, Nd_0.02_Fe_2.98_S_4_, and NFSH. (E) FTIR spectra of Nd_0.02_Fe_2.98_S_4_ and NFSH. (F) Hydrodynamic size distributions of Nd_0.02_Fe_2.98_S_4_ and NFSH. (G) Zeta potentials of Fe_3_S_4_, Nd_0.02_Fe_2.98_S_4_, and NFSH (*n* = 3). High‐resolution XPS spectra of (H) S 2p and (I) Fe 2p in NFSH.

The morphology and structure of Nd_0.02_Fe_2.98_S_4_ and hyaluronic acid‐functionalized Nd_0.02_Fe_2.98_S_4_ nanozymes (NFSH) were first characterized. Transmission electron microscopy (TEM) showed that Nd_0.02_Fe_2.98_S_4_ displayed a compact quasi‐cubic morphology with a nanoscale size of approximately 50 nm (Figure 1A). This morphology was further supported by scanning electron microscopy (SEM), in which abundant cubic‐like nanoparticles with relatively uniform size distribution were observed (Figure S1A). Statistical analysis of 200 particles showed an average particle size of 52.8 ± 1.7 nm (Figure S1B). In the high‐resolution TEM (HRTEM) image, clear lattice fringes were observed, confirming the crystalline nature of the nanoparticles (Figure 1B). The lattice spacing of 0.296 nm was assigned to the (311) plane of Nd_0.02_Fe_2.98_S_4_. In addition, an enlarged interplanar spacing of 0.324 nm was detected in adjacent domains. This lattice expansion was likely caused by the substitutional incorporation of larger Nd^3+^ ions (1 Å) into the iron sulfide lattice, where smaller Fe^3+^ ions (0.64 Å) were partially replaced, resulting in localized lattice distortion [35]. The elemental distribution and composition were then examined to verify the successful incorporation of Nd. High‐angle annular dark‐field scanning TEM (HAADF‐STEM) and elemental mapping showed that Fe, S, Nd, N, and O signals were distributed throughout the nanoparticles (Figure 1C). The homogeneous Fe, S, and Nd signals indicated that Nd was incorporated into the iron sulfide structure without obvious elemental segregation. Energy‐dispersive X‐ray spectroscopy (EDS) further confirmed the presence of Fe, Nd, and S, with Fe and S as the dominant components and Nd as a low‐content dopant (Figure S2). Inductively coupled plasma analysis showed that the Fe, Nd, and S contents were consistent among different NFSH batches. The measured Fe, Nd, and S concentrations were 124.23 ± 1.15, 0.91 ± 0.09, and 167.46 ± 2.15 µmol/L for NFSH‐1; 119.79 ± 2.09, 0.78 ± 0.12, and 168.17 ± 1.97 µmol/L for NFSH‐2; and 123.65 ± 2.11, 0.83 ± 0.10, and 165.91 ± 1.73 µmol/L for NFSH‐3, respectively (Table S1). These results demonstrated the compositional reproducibility of NFSH nanozymes.

The crystal structure was further analyzed by X‐ray diffraction (XRD). The diffraction peaks of Fe_3_S_4_ were well matched with the standard Fe_3_S_4_ pattern (JCPDS No. 16‐0713) [36], confirming the formation of the iron sulfide phase (Figure 1D). After Nd doping and HA modification, Nd_0.02_Fe_2.98_S_4_ and NFSH retained the main diffraction peaks of Fe_3_S_4_, and no obvious impurity peaks were observed. This result suggested that the low‐level Nd dopant and surface modification did not destroy the primary Fe_3_S_4_ crystalline structure. The preserved sulfide framework was important for maintaining magnetic responsiveness and redox‐active catalytic sites, while Nd incorporation was expected to improve the magnetic and optical properties required for magneto‐NIR‐II‐programmed therapy. Fourier‐transform infrared (FTIR) spectroscopy was used to confirm the successful surface functionalization. In the spectra of Nd_0.02_Fe_2.98_S_4_, the characteristic vibration bands related to Fe─S and S─S bonds were observed at 642 and 1076 cm^−1^ [37, 38], respectively (Figure 1E). After HA modification, typical HA‐associated absorption bands appeared in the NFSH spectrum, including C═O stretching at 1613 cm^−1^, COO^−^ vibration at 1482 cm^−1^, and C─N‐related signals in the range of 1200–1400 cm^−1^ [39, 40]. These spectral changes verified that HA was successfully introduced onto the nanoparticle surface. SEM images further showed that NFSH maintained a nanoscale morphology after HA coating, although slight aggregation was observed in the dried state during SEM sample preparation (Figure S3).

Dynamic light scattering analysis also supported the successful surface modification. Compared with Nd_0.02_Fe_2.98_S_4_, NFSH showed an increased hydrodynamic diameter, which changed from approximately 49.6 to 64.3 nm after HA coating (Figure 1F). The polydispersity index increased from 0.183 for Nd_0.02_Fe_2.98_S_4_ to 0.252 for NFSH, indicating a moderate broadening of the size distribution after polymer modification. This increase was consistent with the formation of an HA layer on the nanoparticle surface. The zeta potential also changed markedly during the modification process. Fe_3_S_4_ and Nd_0.02_Fe_2.98_S_4_ showed positive zeta potentials of +22.7 and +20.8 mV, respectively, whereas NFSH showed a negative zeta potential of −12.5 mV (Figure 1G). This charge reversal was attributed to the negatively charged carboxyl groups in HA [41]. The HA‐derived negative surface charge was expected to improve colloidal stability and reduce nonspecific aggregation under physiological conditions. In addition, HA modification provided a molecular basis for CD44‐mediated recognition, which was relevant to subsequent BBB transport and GBM‐targeted accumulation.

The surface elemental composition and chemical valence states of NFSH were further investigated by X‐ray photoelectron spectroscopy (XPS). The XPS survey spectrum showed the coexistence of Nd, Fe, S, N, C, and O, confirming the integration of the Nd‐doped iron sulfide core with HA‐associated surface components (Figure S4). In the high‐resolution S 2p spectrum, deconvoluted peaks corresponding to sulfur species were observed (Figure 1H). The peaks located in the lower binding‐energy region were assigned to metal–sulfur bonding, whereas the peaks at higher binding energies were associated with surface‐oxidized sulfur species [36, 42]. These results indicated that sulfide components were preserved on the NFSH surface. In the Fe 2p spectrum, the peaks at 707.4 and 719.6 eV were assigned to Fe^2+^, while those at 709.6 and 721.3 eV were attributed to Fe^3+^ [19] (Figure 1I). The calculated Fe^2+^/Fe^3+^ ratio was 0.41:0.59, suggesting the coexistence of Fe^2+^ and Fe^3+^ on the NFSH surface. This mixed‐valence Fe state was favorable for electron transfer and redox cycling, which provided an important chemical basis for the cascade nanozyme activity of NFSH, including glutathione consumption and ROS generation. The colloidal stability of NFSH was finally assessed in H_2_O, phosphate‐buffered saline (PBS), and Dulbecco's modified Eagle medium containing 10% fetal bovine serum (DMEM with 10% FBS). The hydrodynamic diameter of NFSH showed only slight fluctuations during storage for 7 days, and no continuous size increase was observed (Figure S5). NFSH remained approximately 60–75 nm in H_2_O, 65–80 nm in PBS, and around 100 nm in DMEM with 10% FBS throughout the observation period. The slightly larger hydrodynamic size in serum‐containing medium was likely caused by protein adsorption on the HA‐modified surface. These results indicated that NFSH possessed sufficient colloidal stability under physiologically relevant conditions.

### 2.2 Magnetic Responsiveness and NIR‐II Optical Properties of NFSH Nanozymes

**FIGURE 2:**
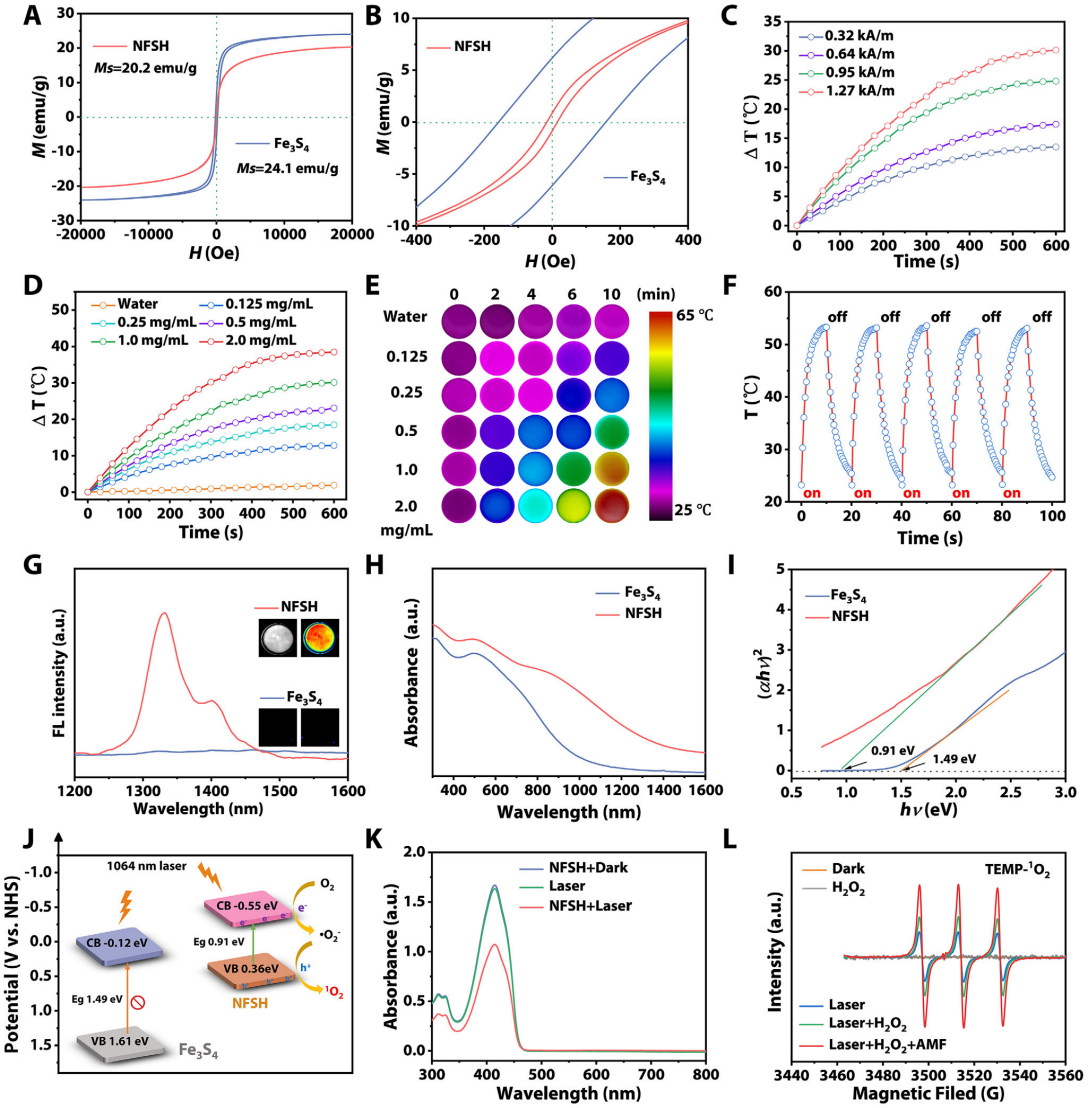
Magnetic and optical properties of NFSH nanozymes. (A) Hysteresis loops of Fe_3_S_4_ and NFSH measured at 300 K. (B) Enlarged hysteresis curves in the magnetic field range from −400 to 400 Oe. (C) Temperature elevation curves of NFSH aqueous solution under AMF treatment at different magnetic field intensities. (D) Concentration‐dependent heating profiles and (E) infrared thermal images of NFSH under AMF treatment. (F) Magnetothermal cycling curve of NFSH under repeated AMF on/off treatment. (G) Emission spectra of Fe_3_S_4_ and NFSH under 808 nm laser excitation; inset shows the corresponding NIR‐II fluorescence image collected with a 1175 nm long‐pass filter. H) UV–vis–NIR absorption spectra and I) bandgap plots of Fe_3_S_4_ and NFSH. (J) Schematic illustration of singlet oxygen (^1^O_2_) generation by NFSH under 1064 nm laser irradiation. (K) UV–vis absorption spectra of DPBF solution with or without NFSH under dark conditions or 1064 nm laser irradiation for 10 min. (L) ESR spectra of TEMP‐trapped ^1^O_2_ under different treatments. Unless otherwise indicated, NFSH was used at 200 µg/mL, H_2_O_2_ at 0.1 mm, the 1064 nm laser at 0.33 W/cm^2^ for 10 min, and AMF at 495 kHz and 1.27 × 10^3^ A/m for 10 min.

The magnetic responsiveness of NFSH nanozymes was systematically evaluated under external magnetic fields. Pristine Fe_3_S_4_ nanoparticles displayed typical ferromagnetic behavior [43, 44], with a saturation magnetization (*M*_s_) of 24.1 emu/g and a coercivity (*H*_c_) of approximately 158.7 Oe (Figure 2A,B). After Nd^3+^ doping and HA modification, the obtained NFSH nanozymes showed a superparamagnetic‐like magnetic profile. Although the M_s_ value was slightly reduced to 20.2 emu/g, the coercivity and remanence were markedly decreased, with an *H*_c_ value of 12.6 Oe. This decrease in magnetization was mainly associated with the incorporation of nonmagnetic/weakly magnetic components and the HA coating layer. Meanwhile, the sharp reduction in coercivity was attributed to Nd^3+^‐mediated modulation of the magnetic lattice and magnetocrystalline anisotropy, which lowered the energy barrier for magnetization reversal and enabled rapid magnetic response after removal of the external field [35, 45]. This magnetic behavior was further confirmed by the magnetic collection experiment. When a 40 mT magnet was placed beside the NFSH aqueous dispersion, the dispersed nanozymes were rapidly attracted toward the magnet‐facing side within 10 s (Figure S6). These findings suggested that NFSH could respond effectively to external magnetic guidance, which was important for subsequent OMF‐assisted BBB penetration and tumor accumulation.

The AMF‐triggered magnetothermal performance of NFSH was then examined. Under AMF treatment, the temperature elevation of the NFSH dispersion increased with magnetic field intensity (Figure 2C). At 1 mg/mL, the temperature rise reached approximately 13.5°C, 17.5°C, 24.5°C, and 30.7°C after 600 s under magnetic field intensities of 0.32, 0.64, 0.95, and 1.27 kA/m, respectively. This field‐dependent heating behavior indicated that magnetic energy was efficiently converted into thermal energy by NFSH. Consistently, the specific absorption rate (SAR) values increased as the magnetic field intensity was enhanced, and the highest SAR value reached 2792.8 W/g under AMF at 495 kHz and 1.27 kA/m (Figure S7), confirming the strong magnetothermal conversion capability of NFSH [46]. The concentration‐dependent heating behavior was further investigated (Figure 2D,E). After AMF exposure for 10 min, pure water exhibited only a slight temperature increase of 1.7°C, whereas NFSH dispersions showed progressively enhanced heating as the concentration increased. In particular, the temperature of the 2 mg/mL NFSH dispersion increased by 38.2°C, indicating that the thermal output could be controlled by adjusting the nanozyme concentration. Infrared thermal images visually confirmed this concentration‐dependent magnetothermal effect. In addition, the thermal stability of NFSH was assessed through five repeated AMF on/off cycles (Figure 2F). The temperature repeatedly increased during AMF exposure and decreased after AMF withdrawal, with no obvious attenuation in heating amplitude. These findings indicated that NFSH possessed stable and reversible magnetothermal behavior.

The NIR‐II fluorescence capability of NFSH was subsequently assessed because Nd^3+^ ions are typical luminescent centers that can generate near‐infrared emission in suitable host matrices [47, 48, 49]. Under 808 nm laser excitation, Fe_3_S_4_ showed almost no detectable emission, whereas NFSH exhibited a distinct NIR‐II fluorescence peak centered at 1318 nm (Figure 2G). This emission was assigned to the ^4^F_3/2_→^4^I_13/2_ electronic transition of Nd^3+^ [21, 50]. The corresponding NIR‐II fluorescence image collected with a 1175 nm long‐pass filter further verified the strong imaging signal of NFSH. These results demonstrated that Nd^3+^ doping endowed the Fe_3_S_4_‐based nanozyme with NIR‐II fluorescence capability, which was beneficial for deep‐tissue imaging and real‐time visualization of nanozyme accumulation in GBM.

The optical absorption and band structure of NFSH were further analyzed to clarify its NIR‐II photoresponsive behavior. Compared with Fe_3_S_4_, NFSH showed stronger and broader absorption across the UV–vis–NIR region, especially in the NIR range (Figure 2H). This enhanced absorption was attributed to the optical contribution of Nd^3+^ ions and the modified electronic structure after rare‐earth doping [51]. The bandgaps were determined from the Tauc plots. The bandgap of Fe_3_S_4_ was estimated to be 1.49 eV, whereas that of NFSH was narrowed to 0.91 eV (Figure 2I). Valence‐band XPS analysis further showed that the valence‐band positions of Fe_3_S_4_ and NFSH were 1.61 and 0.36 eV, respectively (Figure S8). Based on these values, the conduction‐band positions were calculated to be −0.12 eV for Fe_3_S_4_ and −0.55 eV for NFSH. These results suggested that Nd^3+^ doping reconstructed the electronic band structure of Fe_3_S_4_, narrowed the bandgap, and made NFSH more responsive to 1064 nm NIR‐II irradiation. As illustrated in Figure 2J, NFSH could be photoactivated under 1064 nm laser irradiation, resulting in the generation of photogenerated electrons and holes. The electrons on the conduction band could react with O_2_ to form •O_2_^−^, which could subsequently participate in ^1^O_2_ generation. In contrast, Fe_3_S_4_ with a wider bandgap was less favorable for excitation under the same NIR‐II irradiation. Therefore, Nd^3+^ doping was considered to be critical for constructing the NIR‐II‐activated photodynamic function of NFSH.

The ^1^O_2_‐generating ability of NFSH was then verified using 1,3‐diphenylisobenzofuran (DPBF) as a chemical probe [52]. As shown in Figure 2K, the characteristic absorbance of DPBF at approximately 415 nm remained almost unchanged in the dark or under laser irradiation alone. In contrast, a marked decrease in DPBF absorbance was observed when the DPBF/NFSH mixture was exposed to 1064 nm laser irradiation for 10 min, confirming NIR‐II‐triggered ^1^O_2_ generation by NFSH. The effect of laser power density was further examined. As the 1064 nm laser power density was increased from 0 to 1 W/cm^2^, the DPBF absorbance decreased progressively (Figure S9A), indicating that ^1^O_2_ production was enhanced by stronger NIR‐II excitation. Notably, this photodynamic process was further strengthened in the presence of H_2_O_2_ (Figure S9B). This enhancement was likely related to the catalase‐like activity of NFSH, through which H_2_O_2_ could be decomposed to supply O_2_ for type II photodynamic reactions. The strongest DPBF depletion was observed under the combined treatment of NFSH, 1064 nm laser, H_2_O_2_, and AMF, suggesting that AMF‐induced magnetothermal activation promoted the catalytic cascade and increased the availability of oxygen‐related reactive intermediates. Electron spin resonance (ESR) spectroscopy using 2,2,6,6‐tetramethylpiperidine (TEMP) as the trapping agent further supported this conclusion. As shown in Figure 2L, the characteristic TEMP‐^1^O_2_ triplet signal was weak under dark conditions but became stronger after laser irradiation. The signal was further increased in the presence of H_2_O_2_ and reached the highest intensity under the combined laser/H_2_O_2_/AMF treatment. Taken together, these data demonstrated that NFSH functioned as an efficient NIR‐II‐responsive and magnetically programmable oxidative nanozyme platform.

### 2.3 Multienzyme‐Like Catalytic Activities and H2S Release Behavior of NFSH Nanozymes

**FIGURE 3:**
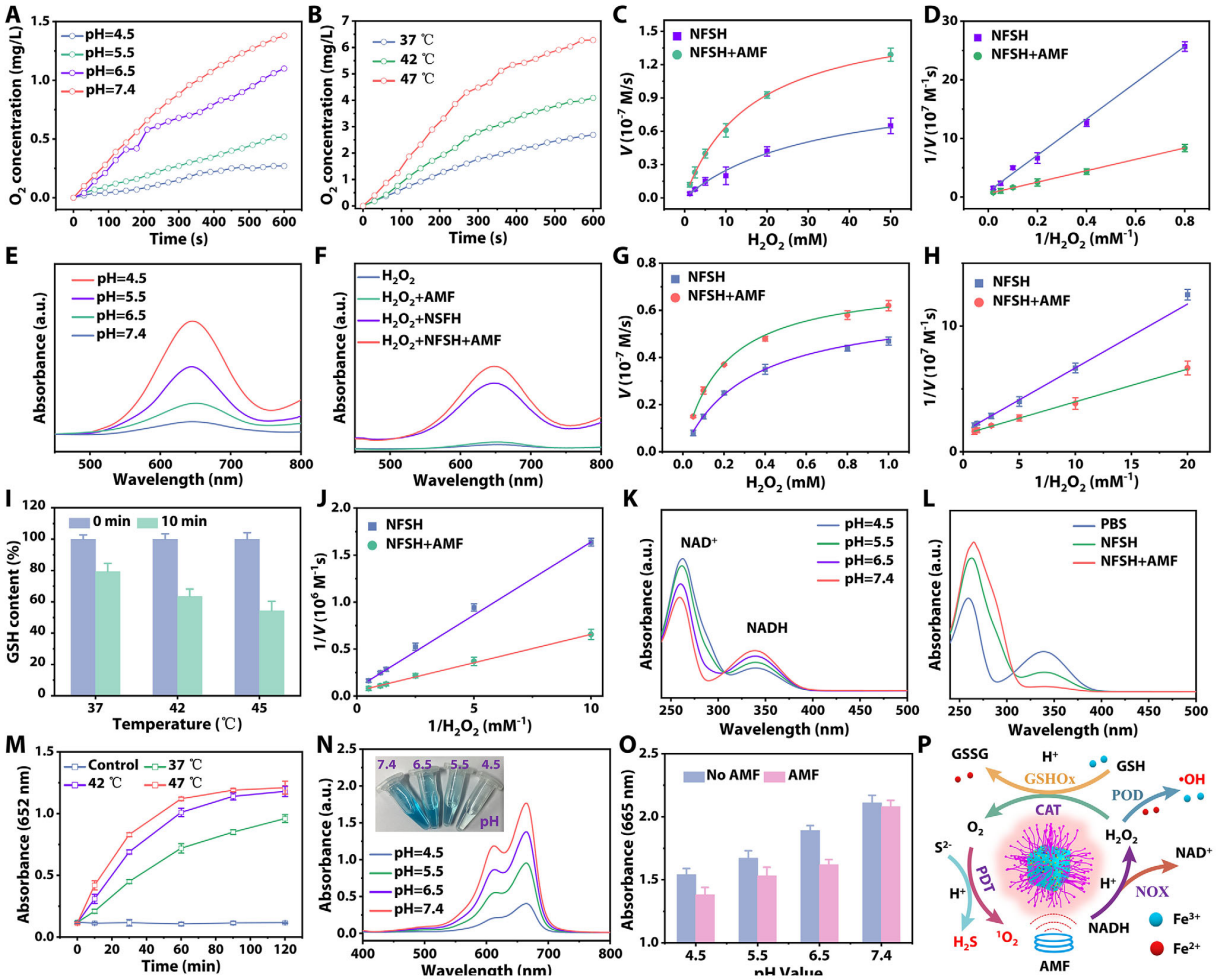
Multienzyme‐like catalytic activity and H_2_S release of NFSH nanozymes. (A) pH‐dependent and (B) temperature‐dependent O_2_ generation in NFSH aqueous solution containing H_2_O_2_. (C) Michaelis–Menten kinetic curves and (D) Lineweaver–Burk plots for the CAT‐like activity of NFSH with or without AMF treatment. (E) pH‐dependent absorbance spectra of oxidized TMB in the presence of NFSH. (F) Absorbance spectra of TMB solution at pH 4.5 under different treatment conditions. (G) Michaelis–Menten kinetic curves and (H) Lineweaver–Burk plots for the POD‐like activity of NFSH with or without AMF treatment. (I) Temperature‐dependent GSH consumption in NFSH‐containing solutions (*n* = 3). (J) Kinetic curves for the GSHOx‐like activity of NFSH with or without AMF treatment. (K) pH‐dependent absorbance spectra of NADH solution containing NFSH. (L) Absorbance spectra of NADH solution containing NFSH with or without AMF treatment. (M) Temperature‐dependent absorbance changes at 652 nm in TMB solution containing NFSH and NADH. (N) pH‐dependent H_2_S release from NFSH detected using MB after 120 min; inset shows the corresponding color changes. (O) Absorbance at 665 nm of MB solutions at different pH values with or without AMF treatment for 10 min (*n* = 3). (P) Schematic illustration of magnetothermal‐regulated catalytic reactions, photodynamic activity, and H_2_S release. Unless otherwise indicated, NFSH was used at 200 µg/mL, H_2_O_2_ at 0.1 mm, AMF at 495 kHz and 1.27 × 10^3^ A/m for 10 min, and H_2_S release assays were performed for 120 min.

The multienzyme‐like catalytic activities of NFSH nanozymes were systematically evaluated, including CAT‐like, POD‐like, GSHOx‐like, and NOX‐like activities. First, the CAT‐like activity was examined by incubating NFSH with H_2_O_2_ and monitoring dissolved O_2_ generation. O_2_ production was increased with increasing pH, and the highest O_2_ level was detected at pH 7.4 (Figure 3A). This result indicated that NFSH could catalyze H_2_O_2_ decomposition to relieve hypoxia under physiological or weakly acidic conditions. Concentration‐dependent experiments further showed that O_2_ generation was enhanced with increasing NFSH and H_2_O_2_ concentrations (Figure S10), confirming that the catalytic process was jointly regulated by nanozyme dose and substrate availability. The influence of magnetothermal activation on CAT‐like activity was further investigated by simulating AMF‐induced temperature elevation. As the temperature was increased from 37°C to 47°C, O_2_ accumulation was markedly elevated (Figure 3B), suggesting that magnetothermal activation promoted H_2_O_2_ decomposition. Michaelis–Menten kinetic curves and Lineweaver–Burk plots further supported this conclusion, as AMF treatment increased the CAT‐like catalytic rate of NFSH toward H_2_O_2_ and improved the apparent enzymatic efficiency (Figure 3C,D).

The POD‐like activity of NFSH was then assessed using 3,3′,5,5′‐tetramethylbenzidine (TMB) as a chromogenic substrate. In the presence of H_2_O_2_, oxidized TMB showed a characteristic absorbance peak at 652 nm. The absorbance intensity was strongly pH‐dependent and increased as the pH decreased from 7.4 to 4.5 (Figure 3E), indicating that acidic conditions favored •OH generation by NFSH. This feature was consistent with the acidic tumor microenvironment and suggested that NFSH could preferentially amplify oxidative stress in tumor‐associated acidic regions. The POD‐like activity was also dependent on the NFSH concentration, and obvious TMB oxidation was observed even at 12.5 µg/mL (Figure S11A). In addition, the absorbance intensity increased with increasing H_2_O_2_ concentration, indicating substrate‐dependent POD‐like catalysis (Figure S11B). Compared with H_2_O_2_ alone and H_2_O_2 _+ AMF, the H_2_O_2 _+ NFSH group showed much stronger TMB oxidation, whereas the H_2_O_2 _+ NFSH + AMF group produced the highest absorbance signal (Figure 3F). This result confirmed that AMF stimulation further enhanced the POD‐like catalytic performance of NFSH. The kinetic parameters further demonstrated the high POD‐like catalytic efficiency of NFSH. The apparent *K*_m_ decreased from 0.32 mM for NFSH to 0.19 mm for NFSH + AMF, while *V*_max_ increased from 0.63 to 0.72 µm/s (Figure 3G,H; Table S2). The lower *K*_m_ indicated improved affinity toward H_2_O_2_ after AMF treatment, whereas the higher *V*_max_ reflected accelerated catalytic turnover. The catalytic efficiency (*k*_cat_/*K*_m_) of NFSH + AMF reached 3.31 × 10^8^ s^−1^/m, which was 2.03‐fold higher than that of NFSH, 11.07‐fold higher than that of Fe_3_S_4_ [36], and 1141.38‐fold higher than that of horseradish peroxidase (HRP) [53]. Therefore, the Nd‐doped Fe sulfide nanozyme displayed strong POD‐like activity, and this activity was further amplified by AMF‐triggered magnetothermal effects.

Since GSH is a major intracellular antioxidant that limits ROS accumulation, the GSHOx‐like activity of NFSH was further investigated. GSH consumption was quantified using the 5,5′‐dithiobis(2‐nitrobenzoic acid) (DTNB) colorimetric assay. The GSHOx‐like activity of NFSH was enhanced under acidic conditions, as reflected by the stronger decrease in the DTNB‐related signal at lower pH values (Figure S12A). GSH depletion was also regulated by NFSH concentration, and higher NFSH concentrations caused more pronounced GSH consumption (Figure S12B). Time‐dependent analysis showed that GSH was rapidly depleted in the presence of NFSH, whereas negligible GSH consumption was observed in the control group (Figure S12C). Importantly, AMF treatment accelerated GSH depletion, and nearly complete GSH consumption was achieved within 120 min in the NFSH + AMF group. Temperature‐dependent experiments further confirmed this magnetothermal enhancement, as the residual GSH content after 10 min decreased more strongly at 42°C and 45°C than at 37°C (Figure 3I). Kinetic fitting using GSH as the substrate showed that AMF treatment increased the catalytic rate of GSH oxidation and improved the apparent GSHOx‐like catalytic performance of NFSH (Figure 3J; Figure S12D). These results indicated that NFSH could weaken the antioxidant defense of tumor cells by consuming GSH, thereby creating a redox environment that favored ROS accumulation and ferroptosis amplification.

The NOX‐like activity of NFSH was subsequently assessed by tracking the conversion of NADH to NAD^+^. As shown in Figure 3K, the characteristic NADH absorbance at around 340 nm decreased progressively as the pH decreased from 7.4 to 4.5, whereas the NAD^+^ signal near 262 nm became more prominent, indicating that acidic conditions promoted NADH oxidation. When AMF was applied, the NADH peak was further suppressed, and the NAD^+^ peak was further intensified (Figure 3L), demonstrating that magnetothermal stimulation accelerated the NOX‐like reaction. Since NADH oxidation has been reported to generate H_2_O_2_ [54, 55], TMB was added to a mixed solution containing NFSH and NADH in the absence of externally supplied H_2_O_2_. The absorbance at 652 nm gradually increased over time, confirming endogenous H_2_O_2_ generation during NADH oxidation (Figure 3M). Moreover, this absorbance increase was more pronounced at elevated temperatures, indicating that thermal activation accelerated H_2_O_2_ production. Therefore, NFSH‐mediated NOX‐like catalysis not only depleted NADH but also supplied H_2_O_2_ for subsequent POD‐like reactions.

To clarify the origin of the enhanced catalytic performance under acidic and magnetothermal conditions, Fe ion release from NFSH was measured by inductively coupled plasma mass spectrometry (ICP‐MS). As shown in Figure S13, Fe ion release was increased at lower pH values and higher temperatures. Under pH 4.5, the Fe release rate increased from approximately 18% at 37°C to more than 30% at 47°C. In contrast, only limited Fe release was observed under near‐neutral conditions. This acid‐ and heat‐responsive Fe release behavior was consistent with the enhanced POD‐like and GSHOx‐like activities of NFSH under acidic and AMF‐stimulated conditions. These results suggested that magnetothermal activation promoted catalytic ion availability, thereby strengthening redox cycling and ROS‐related catalytic reactions.

The H_2_S release behavior of NFSH was then evaluated using the methylene blue (MB) chromogenic method. As shown in Figure 3N, the absorbance of MB at 665 nm decreased as the pH decreased, indicating increased H_2_S release under acidic conditions. Compared with pH 7.4, the absorbance at 665 nm decreased by 18.5%, 46.7%, and 77.3% at pH 6.5, 5.5, and 4.5, respectively. The corresponding color changes further supported the acid‐responsive H_2_S release behavior. In addition, H_2_S release increased with increasing NFSH concentration, as reflected by the concentration‐dependent decrease in MB absorbance and the gradual fading of solution color (Figure S14). These results indicated that NFSH could serve as an acid‐activated H_2_S‐releasing nanozyme in tumor‐relevant environments. AMF stimulation further promoted H_2_S release from NFSH. As shown in Figure 3O, MB absorbance at 665 nm was lower in the AMF‐treated groups than in the non‐AMF groups at acidic pH values, indicating increased H_2_S release after 10 min of AMF exposure. Temperature‐dependent assays also showed that the MB absorption signal decreased as the temperature increased from 37°C to 47°C (Figure S15), further verifying that magnetothermal activation accelerated H_2_S release. Taken together, NFSH was shown to function as a magnetothermal‐regulated cascade nanozyme with CAT‐like, POD‐like, GSHOx‐like, and NOX‐like activities, as well as acid‐ and heat‐responsive H_2_S release behavior (Figure 3P). Under AMF stimulation, NFSH promoted O_2_ production, accelerated H_2_O_2_ conversion into •OH, consumed GSH, oxidized NADH, and enhanced H_2_S release. Such a cascade catalytic profile was considered highly favorable for ferroptosis amplification and for the subsequent disruption of autophagic resistance in GBM.

### 2.4 In Vitro Cellular Uptake, Antitumor Evaluation, and OMF‐Assisted BBB Penetration of NFSH Nanozymes

**FIGURE 4:**
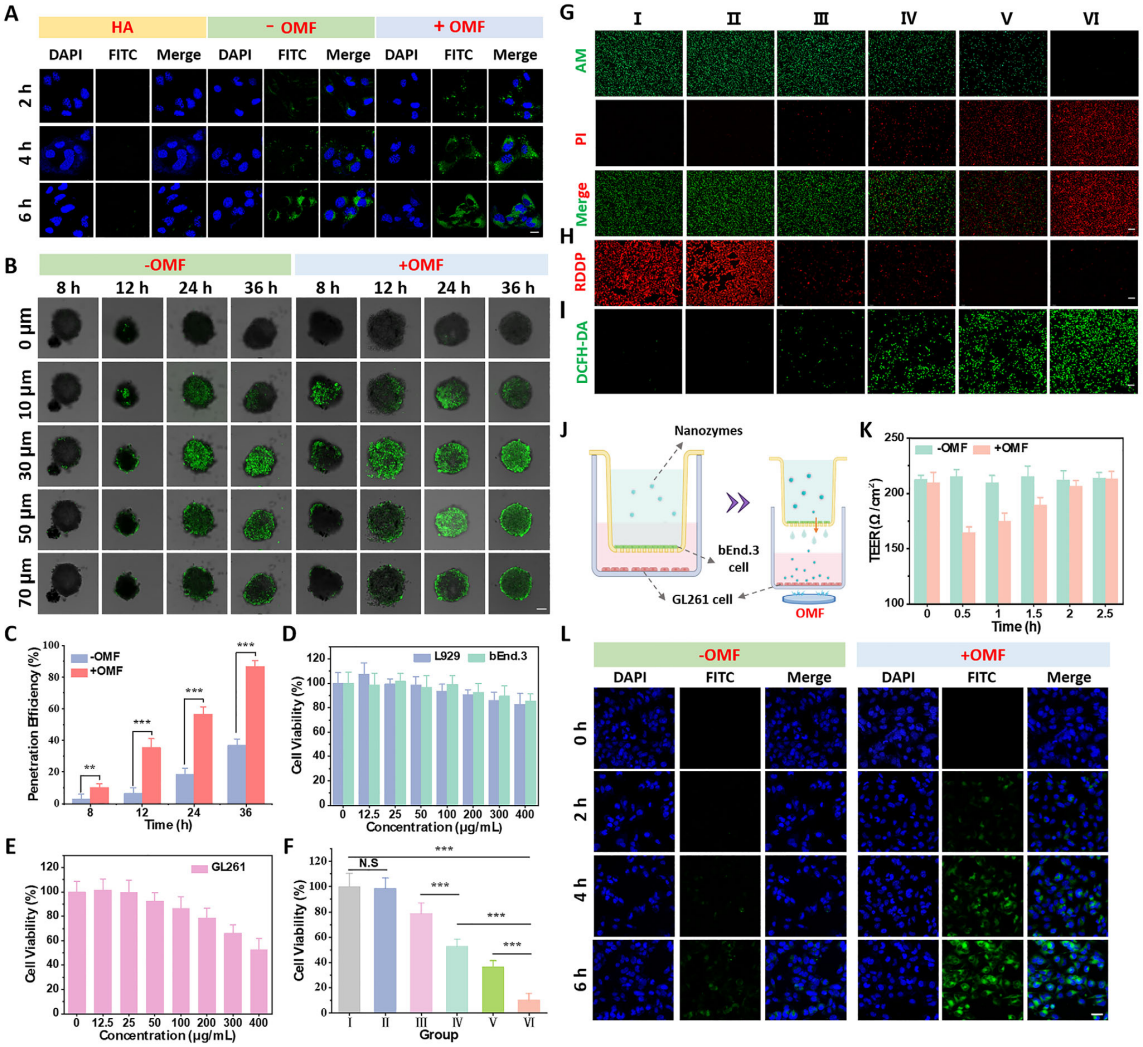
In vitro antitumor activity and BBB penetration of NFSH nanozymes. (A) Confocal laser scanning microscopy (CLSM) images showing NFSH uptake by GL261 cells after different treatments; scale bar: 20 µm. (B) CLSM images and (C) quantitative penetration efficiency of NFSH in MTSs; scale bar: 100 µm; *n *= 3. Cytotoxicity of NFSH toward (D) L929 and bEnd.3 cells and (E) GL261 cells at different concentrations (*n* = 5). (F) Viability of GL261 cells after different treatments (*n* = 5). (G) Calcein‐AM/PI staining, (H) RDPP hypoxia‐probe staining, and (I) DCFH‐DA ROS‐probe staining of GL261 cells after different treatments; scale bars: 50 µm for G and H, and 100 µm for I. (J) Schematic illustration of the in vitro BBB transwell model. (K) TEER of bEnd.3 monolayers in the upper chamber with or without OMF treatment. (L) CLSM images showing NFSH uptake by GL261 cells in the lower chamber; scale bar: 20 µm. Treatment groups were I: PBS, II: AMF + 1064 nm laser, III: NFSH, IV: NFSH + 1064 nm laser, V: NFSH+AMF, and VI: NFSH + 1064 nm laser + AMF. Unless otherwise indicated, NFSH was used at 200 µg/mL, the 1064 nm laser at 0.33 W/cm^2^ for 10 min, AMF at 495 kHz and 1.27 × 10^3^ A/m for 10 min, and OMF was provided by a cylindrical Nd‐Fe‐B magnet with a diameter of 2 cm, thickness of 1 cm, and magnetic field strength of 40 mT. Data are expressed as means ± SD. **p < 0.01, ***p < 0.001. N.S., no significant difference.

Effective cellular uptake was considered a key prerequisite for NFSH‐mediated antitumor therapy. FITC‐labeled NFSH was therefore used to evaluate the internalization behavior of the nanozymes in GL261 cells. As shown in Figure 4A, only weak FITC fluorescence was observed in HA‐pretreated GL261 cells, even after prolonged incubation. This result suggested that free HA competitively blocked CD44 receptors on the GL261 cell membrane and reduced HA‐mediated recognition. In contrast, clear intracellular green fluorescence was detected in GL261 cells treated with NFSH without HA blockade, and the fluorescence signal gradually increased from 2 to 6 h. These findings indicated that HA modification promoted CD44‐mediated cellular uptake of NFSH. Notably, stronger FITC fluorescence was observed after OMF treatment at each time point. This enhancement was attributed to the magnetic responsiveness of NFSH, which allowed OMF‐driven enrichment around tumor cells and accelerated cellular internalization. Thus, NFSH achieved efficient GL261 cell uptake through the combined effects of HA‐mediated molecular targeting and magnetic‐field‐assisted accumulation.

The tumor penetration ability of NFSH was further evaluated using GL261 multicellular tumor spheroids (MTSs), which provided a compact three‐dimensional model to mimic the diffusion barriers of solid GBM tissues. As shown in Figure 4B, weak fluorescence was detected in the inner region of MTSs at early time points without OMF, indicating limited passive diffusion. After incubation for 24 and 36 h, FITC fluorescence gradually extended from the peripheral region toward deeper areas of the spheroids. However, the penetration depth remained limited, reaching only approximately 30–50 µm after 36 h. In comparison, OMF treatment markedly promoted the inward transport of NFSH. Stronger and more widely distributed green fluorescence was observed throughout the spheroids, especially after 24 and 36 h. Quantitative analysis further confirmed this trend (Figure 4C). The penetration efficiency was significantly increased under OMF and reached 82.3% after 36 h, whereas the efficiency without OMF was much lower. These results demonstrated that OMF effectively enhanced the penetration of NFSH into dense tumor‐like structures.

The cytocompatibility of NFSH was then assessed in normal L929 fibroblasts and bEnd.3 brain endothelial cells by the MTT assay. As shown in Figure 4D, both cell types maintained high viability after incubation with NFSH over a concentration range of 12.5–400 µg/mL. Even at 400 µg/mL, cell viability remained above 80%, indicating that NFSH showed acceptable biosafety toward normal cells under the tested conditions. In contrast, NFSH caused a concentration‐dependent decrease in GL261 cell viability (Figure 4E). This selective cytotoxicity was likely related to the tumor microenvironment‐responsive catalytic behavior of NFSH. The relatively acidic microenvironment, elevated H_2_O_2_ levels, and abundant intracellular GSH in tumor cells could activate cascade catalytic reactions, leading to oxidative stress and tumor cell damage. Therefore, NFSH displayed a degree of tumor‐selective toxicity while maintaining limited toxicity toward normal cells. The antitumor activity of NFSH was further examined under 1064 nm laser irradiation and AMF stimulation. As shown in Figure 4F, AMF plus 1064 nm laser treatment alone did not induce obvious cytotoxicity, and GL261 cell viability remained close to that of the PBS group. This result confirmed that the external stimuli were safe under the applied conditions. The material‐alone group reduced GL261 cell viability to approximately 80%, indicating moderate tumor inhibition. When NFSH was combined with 1064 nm laser irradiation, cell viability was further decreased, suggesting that NIR‐II‐triggered photodynamic activity contributed to tumor cell killing. AMF stimulation produced a stronger inhibitory effect than laser irradiation alone, which may be associated with AMF‐enhanced catalytic reactions and magnetic activation. The most pronounced cytotoxicity was observed in the NFSH + 1064 nm laser + AMF group, in which GL261 cell viability decreased to only 8.9%. These results demonstrated that NIR‐II irradiation and AMF acted cooperatively to amplify the antitumor activity of NFSH.

Calcein‐AM/PI staining provided direct visual evidence of the therapeutic effect. As shown in Figure 4G, intense green fluorescence and minimal red fluorescence were observed in the PBS and AMF + 1064 nm laser groups, indicating that most cells remained viable. In the material‐alone group, PI‐positive cells increased, suggesting that NFSH induced partial cell death. Stronger red fluorescence was observed after either 1064 nm laser irradiation or AMF stimulation, while the most extensive PI staining was detected in the NFSH + 1064 nm laser + AMF group. In this group, the green Calcein‐AM signal was markedly reduced, and dense red PI fluorescence was observed, indicating severe membrane damage and extensive tumor cell death. To clarify the mechanism underlying the synergistic therapeutic effect, intracellular hypoxia and ROS generation were further evaluated. [Ru(dpp)_3_]Cl_2_ (RDPP) staining was first used to assess intracellular O_2_ levels, because stronger RDPP fluorescence indicated a more hypoxic state. As shown in Figure 4H, strong red fluorescence was detected in the PBS and AMF + 1064 nm laser groups, suggesting severe intracellular hypoxia. After NFSH treatment, the RDPP signal was reduced, indicating that NFSH partially relieved hypoxia through catalase‐like O_2_ generation. The fluorescence signal further decreased after AMF stimulation, which suggested that AMF enhanced the catalytic oxygen‐producing ability of NFSH. Although 1064 nm laser irradiation could consume O_2_ during photodynamic therapy, the RDPP signal in the NFSH + 1064 nm laser group remained lower than that in the control groups. This result indicated that NFSH‐mediated O_2_ generation partly compensated for O_2_ consumption and improved the hypoxic microenvironment. The weakest RDPP fluorescence was observed in the NFSH + 1064 nm laser + AMF group, confirming that dual‐stimulus activation effectively relieved intracellular hypoxia. Intracellular ROS production was detected using the DCFH‐DA probe. As shown in Figure 4I, almost no green fluorescence was observed in the PBS and AMF + 1064 nm laser groups, indicating negligible ROS generation. In contrast, NFSH‐treated cells showed detectable green fluorescence, confirming that NFSH induced ROS production in GL261 cells. The fluorescence intensity increased after 1064 nm laser irradiation or AMF stimulation, demonstrating that both external triggers enhanced the ROS‐generating ability of NFSH. The brightest DCF fluorescence was observed in the NFSH + 1064 nm laser + AMF group. This result indicated that AMF and NIR‐II irradiation cooperatively promoted ROS amplification. Together with the RDPP results, these findings suggested that NFSH relieved hypoxia while enhancing ROS generation, thereby providing a favorable basis for amplified oxidative tumor damage. The therapeutic performance of NFSH was further validated in GL261 MTSs. As shown in Figure S16, the strongest inhibitory effect was achieved in the NFSH + 1064 nm laser + AMF group, with a tumor inhibition rate of 93.6%. These three‐dimensional results were consistent with the two‐dimensional cell viability data and further demonstrated the cooperative antitumor efficacy of NFSH under NIR‐II laser and AMF stimulation.

Since BBB penetration is a major obstacle for GBM nanotherapy, the trans‐BBB transport ability of NFSH was evaluated using an in vitro BBB transwell model. Previous studies showed that magnetic nanoparticles could be guided across the BBB under magnetic fields [56, 57, 58]. Based on the magnetic responsiveness of NFSH, a transwell system was constructed with bEnd.3 cells in the upper chamber and GL261 cells in the lower chamber (Figure 4J). When the transepithelial/transendothelial electrical resistance (TEER) value of the bEnd.3 monolayer reached approximately 200 Ω/cm^2^, the BBB model was considered successfully established. FITC‐labeled NFSH was then added to the upper chamber, and a cylindrical Nd‐Fe‐B magnet was placed below the lower chamber to provide an OMF. As shown in Figure 4K, the TEER value remained stable at approximately 210 Ω/cm^2^ in the absence of OMF, indicating that the endothelial barrier was maintained. After OMF exposure, the TEER value transiently decreased to approximately 165 Ω/cm^2^ at 0.5 h and then gradually recovered to the initial level within 2–2.5 h. This reversible TEER change suggested that OMF temporarily increased BBB permeability without causing persistent endothelial barrier damage. CLSM images of GL261 cells in the lower chamber further confirmed OMF‐assisted transport (Figure 4L). Without OMF, only weak FITC fluorescence was detected in GL261 cells, even after 6 h of incubation. In contrast, the OMF‐treated group showed a clear time‐dependent increase in intracellular FITC fluorescence from 2 to 6 h. These results indicated that OMF promoted the transport of NFSH across the bEnd.3 monolayer and enhanced subsequent uptake by GL261 cells.

### 2.5 In Vitro Evaluation of Mitochondrial Function, Autophagy Modulation, Ferroptosis‐Related Markers, and Macrophage Polarization

**FIGURE 5:**
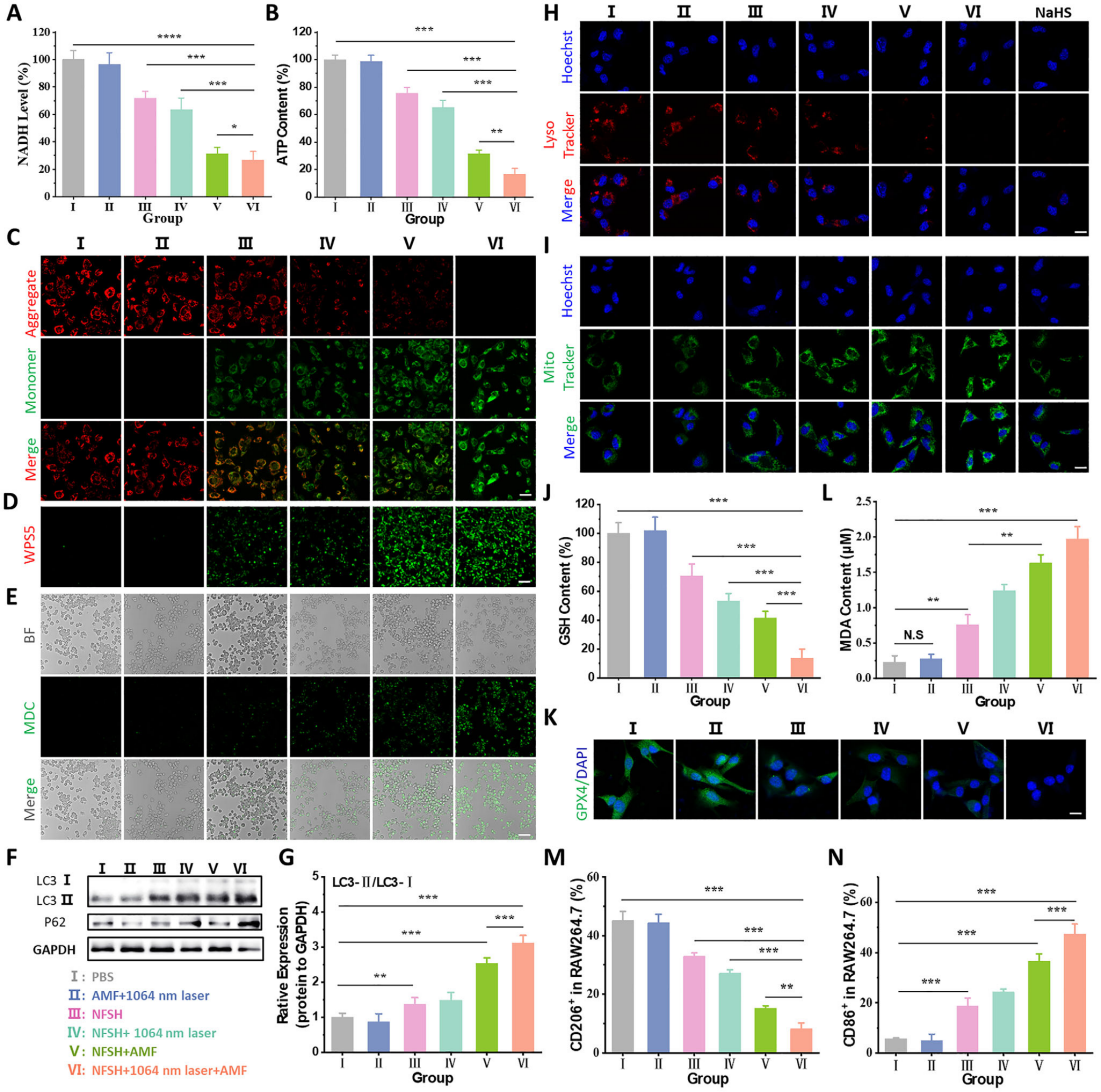
In vitro analysis of mitochondrial function, autophagy, ferroptosis‐related markers, and macrophage polarization after NFSH treatment. Intracellular (A) NADH levels and (B) ATP contents in GL261 cells after different treatments. (C) CLSM images of JC‐1‐stained GL261 cells after different treatments; scale bar: 20 µm. (D) Intracellular H_2_S fluorescence images in GL261 cells after different treatments; scale bar: 50 µm. (E) CLSM images of MDC‐stained GL261 cells after different treatments; scale bar: 100 µm. (F) Western blot images of LC3‐I/II and P62 expression in GL261 cells after different treatments. (G) Quantitative analysis of the LC3‐II/I ratio from panel F. CLSM images of (H) Lyso‐Tracker‐stained and (I) Mito‐Tracker‐stained GL261 cells after different treatments; scale bar: 20 µm. (J) Intracellular GSH contents after different treatments. (K) CLSM images showing GPX4 expression in GL261 cells after different treatments; scale bar: 20 µm. (L) MDA contents after different treatments. Flow cytometry‐based proportions of (M) M1‐type and (N) M2‐type TAMs after different treatments. Treatment groups were I: PBS, II: AMF + 1064 nm laser, III: NFSH, IV: NFSH + 1064 nm laser, V: NFSH + AMF, and VI: NFSH + 1064 nm laser + AMF. Unless otherwise indicated, NFSH was used at 200 µg/mL, the 1064 nm laser at 0.33 W/cm^2^ for 10 min, and AMF at 495 kHz and 1.27 × 10^3^ A/m for 10 min. Data are presented as means ± SD (*n* = 3). *p < 0.05, **p < 0.01, ***p < 0.001. N.S., not significant.

The intracellular metabolic disturbance caused by NFSH was first investigated by measuring NADH and ATP levels in GL261 cells. Because NFSH exhibited NADH‐consuming oxidase‐like activity, intracellular NADH was used as a key indicator of redox metabolism. As shown in Figure 5A, no obvious decrease in NADH was observed in the AMF + 1064 nm laser group compared with the PBS group, which indicated that the physical stimuli alone caused minimal metabolic interference. By contrast, NADH was markedly depleted after NFSH treatment. This effect was further strengthened by external stimulation, especially in the NFSH + AMF and NFSH + 1064 nm laser + AMF groups. The lowest NADH level was detected after dual AMF/NIR‐II activation, suggesting that magneto‐NIR‐II co‐stimulation accelerated the NOX‐like catalytic process and intensified redox cofactor exhaustion. Consistent with NADH depletion, ATP production was also suppressed after NFSH treatment. As displayed in Figure 5B, the ATP contents in the NFSH, NFSH + 1064 nm laser, NFSH + AMF, and NFSH + 1064 nm laser + AMF groups decreased by 21.3%, 34.6%, 69.4%, and 83.6%, respectively, compared with the PBS group. These results indicated that AMF activation contributed more strongly to ATP deprivation than 1064 nm laser irradiation alone. The strongest ATP reduction was observed under dual AMF and NIR‐II laser stimulation, which supported the synergistic disruption of mitochondrial energy metabolism.

Mitochondrial membrane potential was further examined by JC‐1 staining. In healthy mitochondria, JC‐1 formed aggregates and emitted red fluorescence, whereas mitochondrial depolarization promoted the formation of JC‐1 monomers with green fluorescence [59]. As indicated in Figure 5C, strong red fluorescence and weak green fluorescence were observed in the PBS and AMF + 1064 nm laser groups, indicating that mitochondrial membrane potential was well maintained in the absence of NFSH. After NFSH treatment, red fluorescence was weakened, and green fluorescence was increased, confirming mitochondrial depolarization. This transition became more evident after single‐stimulus activation and was most pronounced in the NFSH + 1064 nm laser + AMF group. These JC‐1 results were consistent with the NADH and ATP assays and demonstrated that NFSH‐mediated NOX‐like catalysis, especially under magneto‐NIR‐II activation, induced severe mitochondrial dysfunction in GL261 cells.

Intracellular H_2_S generation was then detected using the WPS‐5 fluorescent probe. As depicted in Figure 5D, only weak fluorescence was observed in the PBS and AMF + 1064 nm laser groups. In contrast, a clear green fluorescence signal appeared in NFSH‐treated cells, indicating intracellular H_2_S release. The fluorescence was further intensified in the NFSH + AMF and NFSH + 1064 nm laser + AMF groups, suggesting that AMF‐derived thermal activation promoted H_2_S release from NFSH. The dual‐stimulus group displayed the strongest H_2_S fluorescence, which indicated that magneto‐NIR‐II activation enhanced H_2_S generation in the intracellular microenvironment. Autophagosome accumulation was assessed by monodansylcadaverine (MDC) staining. As demonstrated in Figure 5E, weak MDC fluorescence was detected in the PBS and AMF + 1064 nm laser groups, whereas NFSH‐treated cells showed markedly enhanced green fluorescence. This result indicated that autophagosomes accumulated after NFSH treatment. The fluorescence intensity increased further after 1064 nm laser or AMF activation and reached the highest level in the NFSH + 1064 nm laser + AMF group. Since autophagosome accumulation may result from either autophagy activation or blocked autophagic degradation, western blotting was performed to clarify the status of autophagic flux. The expression levels of microtubule‐associated protein 1 light chain 3 (LC3) and sequestosome 1 (P62) were analyzed as representative autophagy‐related markers. During autophagosome formation, LC3‐I was lipidated into LC3‐II, whereas impaired autophagic flux caused abnormal accumulation of LC3‐II and P62 [60, 61]. As presented in Figure 5F,G, the LC3‐II/LC3‐I ratio was increased after NFSH treatment and was further elevated after physical‐field stimulation. The highest LC3‐II/LC3‐I ratio was detected in the NFSH + 1064 nm laser + AMF group. In parallel, P62 expression was also increased, and the highest P62 level was observed in the dual‐stimulus group (Figure S17). The simultaneous accumulation of LC3‐II and P62 indicated that NFSH did not simply activate autophagy initiation but blocked autophagic degradation after autophagosome formation. This blockade was likely associated with H_2_S‐mediated lysosomal disruption and ATP deficiency caused by mitochondrial metabolic failure.

To further verify the impairment of lysosome‐dependent degradation, lysosomal acidification was evaluated by Lyso‐Tracker staining. As displayed in Figure 5H and Figure S18, intense red fluorescence was observed in the PBS and AMF + 1064 nm laser groups, suggesting normal lysosomal acidification. In contrast, Lyso‐Tracker fluorescence was markedly reduced after NFSH treatment, especially in the NFSH + AMF and NFSH + 1064 nm laser + AMF groups. The red fluorescence almost disappeared in the dual‐stimulus group, indicating severe lysosomal dysfunction. A similar reduction in Lyso‐Tracker fluorescence was observed in the NaHS‐treated group, which further supported the role of H_2_S in lysosomal damage. These results demonstrated that NFSH‐induced H_2_S release disrupted lysosomal acidification, thereby preventing the degradation of autophagosomes. Mitochondrial accumulation was further evaluated by Mito‐Tracker staining. As shown in Figure 5I and Figure S19, Mito‐Tracker fluorescence was progressively increased in NFSH‐treated cells, with the strongest signal detected in the NFSH + 1064 nm laser + AMF group. This result indicated that damaged mitochondria accumulated because they could not be efficiently cleared through lysosome‐dependent mitophagy. In contrast, NaHS treatment alone did not cause a comparable increase in mitochondrial abundance, suggesting that H_2_S‐mediated lysosomal impairment was insufficient to induce marked mitochondrial accumulation without NFSH‐induced mitochondrial injury. Taken together, these results demonstrated that NFSH caused mitochondrial damage and, at the same time, blocked mitophagic clearance by combining NADH depletion, ATP deficiency, and H_2_S‐mediated lysosomal dysfunction. This dual action was expected to weaken the autophagy‐mediated repair capacity of GBM cells.

Ferroptosis‐related biochemical markers were subsequently examined to determine whether mitochondrial dysfunction and autophagy blockade were accompanied by lipid peroxidation. Intracellular GSH content was first measured because GSH served as a major antioxidant substrate for GPX4‐mediated lipid peroxide detoxification. As presented in Figure 5J, GSH levels remained high in the PBS and AMF + 1064 nm laser groups, whereas NFSH treatment significantly depleted GSH. The GSH content was further decreased after 1064 nm laser or AMF activation. The most pronounced GSH depletion was observed in the NFSH + 1064 nm laser + AMF group, where the GSH level decreased by 89.7% compared with the PBS group. This result confirmed that magneto‐NIR‐II activation reinforced the glutathione oxidase‐like catalytic cascade of NFSH and severely weakened the intracellular antioxidant defense system. GPX4 expression was then detected by immunofluorescence staining. As exhibited in Figure 5K and Figure S20, strong GPX4 fluorescence was observed in the PBS and AMF + 1064 nm laser groups. After NFSH treatment, GPX4 fluorescence was reduced, indicating suppression of the GPX4‐dependent anti‐ferroptotic pathway. This reduction became more obvious after single‐stimulus activation and was most evident in the NFSH + 1064 nm laser + AMF group, where GPX4 fluorescence was reduced by 91.3%. Since GPX4 is responsible for detoxifying lipid hydroperoxides, its depletion suggested that NFSH treatment sensitized GL261 cells to ferroptotic lipid damage.

Lipid peroxidation was further assessed by measuring malondialdehyde (MDA), a representative terminal product of lipid oxidation. As indicated in Figure 5L, MDA content was only slightly changed in the AMF + 1064 nm laser group compared with the PBS group. In contrast, MDA levels increased markedly after NFSH treatment and continued to rise after physical‐field activation. The NFSH + 1064 nm laser + AMF group showed the highest MDA level, which was approximately 8.3‐fold higher than that of the PBS group. In addition, intracellular Fe^2+^ contents were significantly elevated after NFSH treatment (Figure S21). The increase was more prominent in the AMF‐containing groups, suggesting that magnetic thermal activation promoted Fe^2+^ release or Fe‐related redox cycling. Therefore, the combined increase in Fe^2+^, GSH depletion, GPX4 suppression, and MDA accumulation confirmed that NFSH efficiently amplified ferroptosis under AMF and NIR‐II laser co‐stimulation.

The immunomodulatory effect of NFSH was further investigated because ferroptosis‐associated oxidative stress and metabolic remodeling may affect the TIME. RAW264.7 macrophages were pretreated with interleukin‐4 (IL‐4) to induce an M2‐like phenotype and were then exposed to different treatment conditions. Flow cytometry was used to analyze CD206^+^ M2‐type macrophages and CD86^+^ M1‐type macrophages. As shown in Figure 5M and Figure S22, the proportions of CD206^+^ M2‐like macrophages were 45.1% and 44.3% in the PBS and AMF + 1064 nm laser groups, respectively. These values decreased to 32.9%, 27.1%, 15.3%, and 8.2% in the NFSH, NFSH + 1064 nm laser, NFSH + AMF, and NFSH + 1064 nm laser + AMF groups, respectively. This decrease indicated that NFSH treatment suppressed the M2‐like immunosuppressive phenotype. In contrast, CD86^+^ M1‐like macrophages showed the opposite trend. As shown in Figure 5N and Figure S22, the proportion of CD86^+^ macrophages was only 5.7% in the PBS group and 4.9% in the AMF + 1064 nm laser group. After NFSH treatment, this proportion increased to 18.7%. Further increases were detected in the NFSH + 1064 nm laser and NFSH + AMF groups, reaching 24.3% and 36.6%, respectively. The highest CD86^+^ macrophage proportion was observed in the NFSH + 1064 nm laser + AMF group, reaching 43.7%. These findings demonstrated that NFSH, especially under magneto‐NIR‐II co‐stimulation, promoted macrophage repolarization from the M2‐like phenotype toward the M1‐like phenotype.

### 2.6 In Vivo NIR‐II Fluorescence and T_2_w‐MRI of NFSH Nanozymes

**FIGURE 6:**
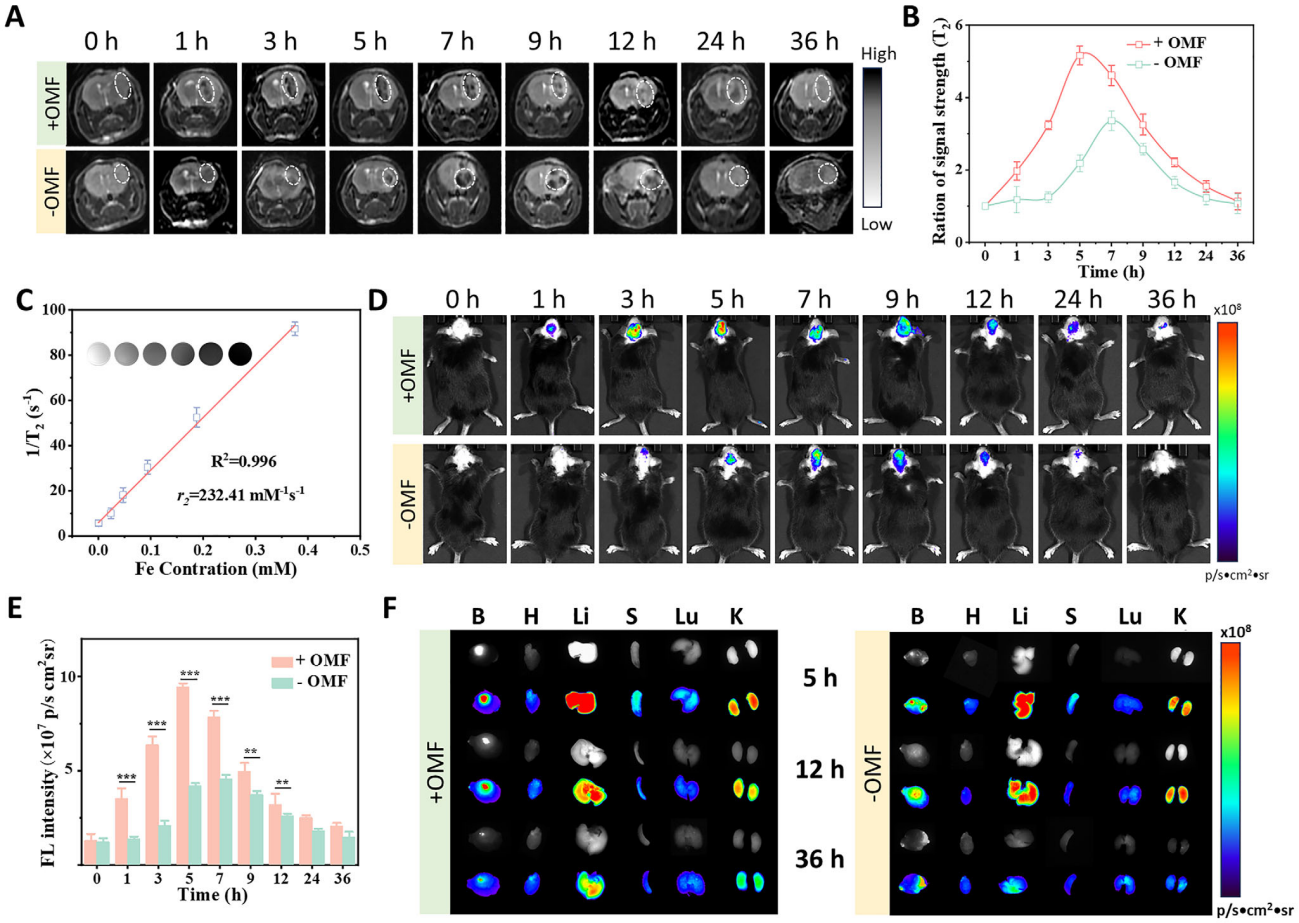
In vivo dual‐modal imaging of orthotopic GBM‐bearing mice after NFSH administration. (A) T_2_‐weighted magnetic resonance (MR) images of the brains of orthotopic GBM‐bearing mice after intravenous injection of NFSH. (B) Quantitative analysis of T_2_ signal intensity at tumor sites from panel A. (C) T_2_ relaxation times of NFSH solutions at different Fe concentrations; inset shows the corresponding T_2_‐weighted MR images. (D) NIR‐II fluorescence images of orthotopic GBM‐bearing mouse brains after intravenous injection of NFSH. (E) Quantitative analysis of fluorescence signals at tumor sites from panel D. (F) Ex vivo NIR‐II fluorescence images of tumors and major organs collected from mice with or without OMF treatment. NFSH was administered at 200 µL and 15 mg/kg. OMF was provided by a cylindrical Nd‐Fe‐B magnet with a diameter of 2 cm, thickness of 1 cm, and magnetic field strength of 40 mT. T: tumor; H: heart; Li: liver; S: spleen; Lu: lung; K: kidney. Data are expressed as means ± SD (*n* = 5). **p < 0.01, ***p < 0.001.

The in vivo dual‐modal imaging performance of NFSH nanozymes was evaluated in orthotopic GBM‐bearing mice by T_2_w‐MRI and NIR‐II fluorescence imaging. After intravenous administration of NFSH, an OMF was applied near the tumor region to guide magnetic accumulation and facilitate trans‐BBB delivery. As exhibited in Figure 6A, time‐dependent darkening of the T_2_w‐MRI signal was observed at the tumor site after NFSH injection, which indicated progressive tumor enrichment of the nanozymes. Quantitative analysis further showed that the T_2_ signal ratio increased more rapidly in the +OMF group than in the −OMF group and reached a maximum at 5 h post‐injection (Figure 6B). The peak T_2_ signal ratio in the +OMF group was approximately 5.2, whereas that in the −OMF group was approximately 3.4. These results suggested that OMF guidance markedly improved the accumulation efficiency of NFSH in the orthotopic GBM region. The time‐dependent MRI profile also showed that OMF treatment prolonged the retention of NFSH at the tumor site. In the +OMF group, clear T_2_ contrast remained visible from 5 to 12 h and was still detectable at 24 h after injection, whereas the signal in the −OMF group decreased more rapidly after reaching its peak. This sustained contrast enhancement was attributed to the combined effects of HA‐mediated CD44 targeting and OMF‐assisted magnetic navigation, which increased BBB permeability and promoted nanozyme deposition within the tumor tissue. These findings supported the use of OMF as an external driving strategy to improve both trans‐BBB delivery and imaging‐guided tumor localization. The transverse relaxation performance of NFSH was further quantified using solutions with different Fe concentrations. As displayed in Figure 6C, the T_2_‐weighted MR signal gradually decreased with increasing Fe concentration, and a strong linear relationship was obtained between 1/*T*_2_ and Fe concentration. The transverse relaxivity (*r*_2_) was calculated to be 232.41 mm ^−1^/s, with an *R* ^2^ value of 0.996. This high *r*_2_ value indicated that NFSH acted as an efficient T_2_‐MRI contrast agent [62, 63, 64]. Therefore, the combination of intrinsic magnetic responsiveness and strong T_2_ relaxation ability enabled NFSH to serve not only as a therapeutic nanozyme but also as an imaging probe for real‐time monitoring of intracranial tumor accumulation.

The NIR‐II fluorescence imaging capability of NFSH was then investigated to provide complementary optical visualization of its in vivo distribution. As indicated in Figure 6D, fluorescence signals at the orthotopic GBM site gradually increased after intravenous injection of NFSH. In the +OMF group, the tumor fluorescence signal became evident at 1 h, increased sharply from 3 to 5 h, and reached its maximum at 5 h post‐injection. Quantitative fluorescence analysis confirmed this trend (Figure 6E). The fluorescence intensity in the +OMF group was significantly higher than that in the −OMF group from 1 to 12 h, with the strongest difference observed at 5h. Based on these imaging results, 5 h post‐injection was selected as the optimal time point for subsequent therapeutic intervention. Ex vivo NIR‐II fluorescence imaging was performed at 5, 12, and 36 h after injection to further assess tissue distribution (Figure 6F). Stronger fluorescence signals were observed in tumors from the +OMF group than in those from the −OMF group at all examined time points, confirming that OMF promoted tumor‐specific accumulation of NFSH. The tumor signal remained detectable even at 36 h in the +OMF group, indicating extended intratumoral retention. In addition, prominent fluorescence signals were detected in the liver and kidneys across all groups, and the signal intensity gradually decreased over time (Figure S23), implying the possible involvement of hepatobiliary and renal metabolism pathways [19, 65]. Therefore, these results demonstrated that NFSH enabled OMF‐enhanced trans‐BBB delivery, tumor‐selective accumulation, and dual‐modal imaging‐guided localization in orthotopic GBM‐bearing mice.

### 2.7 In Vivo Antitumor Evaluation of NFSH Nanozymes in Orthotopic GBM‐Bearing Mice

**FIGURE 7:**
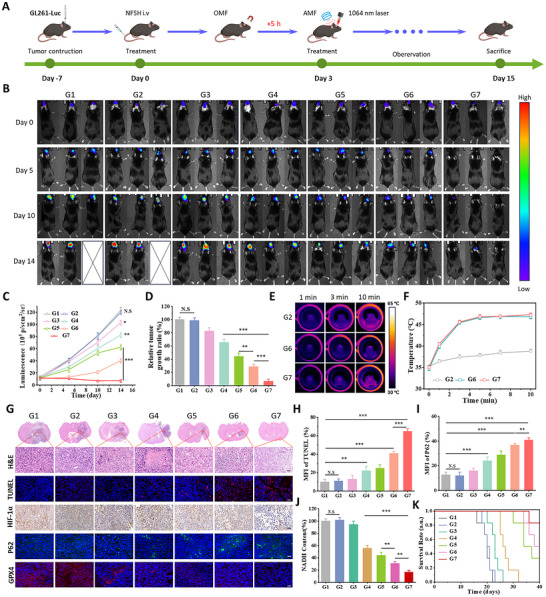
In vivo antitumor evaluation of NFSH nanozymes in orthotopic GBM‐bearing mice. (A) Schematic illustration of the treatment schedule for NFSH‐mediated GBM therapy. (B) Bioluminescence images of GL261‐bearing mice during the treatment period. (C) Quantitative analysis of bioluminescence signals from panel B (*n* = 6). (D) Relative tumor growth ratios in different treatment groups (*n* = 6). (E) Infrared thermal images of GL261‐bearing mice during AMF treatment. (F) Temperature changes at tumor sites during AMF treatment. (G) H&E, TUNEL, HIF‐1α, P62, and GPX4 staining of tumor sections from different treatment groups; scale bar: 100 µm. Quantitative fluorescence intensity analysis of (H) TUNEL and (I) P62 staining in tumor sections from different treatment groups. (J) NADH contents in tumor tissues after different treatments (*n* = 5). (K) Survival curves of mice in different treatment groups (*n* = 6). Treatment groups were G1: PBS, G2: AMF + 1064 nm laser, G3: NFSH, G4: NFSH + OMF, G5: NFSH + OMF + 1064 nm laser, G6: NFSH + OMF + AMF, and G7: NFSH + OMF + AMF + 1064 nm laser. Unless otherwise indicated, NFSH was administered at 15 mg/kg, the 1064 nm laser was applied at 0.33 W/cm^2^ for 10 min, AMF was applied at 495 kHz and 1.27 × 10^3^ A/m for 10 min, and OMF was applied using a cylindrical Nd‐Fe‐B magnet with a diameter of 2 cm, thickness of 1 cm, and magnetic field strength of 40 mT for 5 h. Data are displayed as mean ± SD. *p < 0.05, **p < 0.01, ***p < 0.001. N.S., not significant.

The in vivo antitumor efficacy of NFSH nanozymes was evaluated in orthotopic GL261‐Luc GBM‐bearing mice according to the treatment schedule shown in Figure 7A. The therapeutic effect was first assessed by longitudinal bioluminescence imaging after intraperitoneal injection of luciferin substrate. As presented in Figure 7B, strong and progressively increased bioluminescence signals were observed in the PBS group (G1), indicating rapid intracranial tumor growth. A similar tumor growth trend was observed in the AMF + 1064 nm laser group without NFSH administration (G2), suggesting that the external stimuli alone produced negligible tumor inhibition under the tested conditions. NFSH alone (G3) slightly delayed tumor progression, whereas NFSH combined with OMF (G4) produced a stronger inhibitory effect. This improvement was attributed to OMF‐assisted magnetic enrichment and HA‐mediated CD44 targeting, which promoted NFSH accumulation in orthotopic GBM tissues. The antitumor effect was further strengthened when external energy stimulation was introduced. Compared with G4, both NFSH + OMF + 1064 nm laser (G5) and NFSH + OMF + AMF (G6) markedly suppressed tumor growth, demonstrating that NIR‐II photoactivation and AMF‐mediated activation each contributed to NFSH‐based therapy. The strongest tumor inhibition was achieved in the NFSH + OMF + AMF + 1064 nm laser group (G7). Quantitative bioluminescence analysis showed that the signal intensity in G7 remained at a low level throughout the treatment period, whereas the signals in G1 and G2 increased sharply with time (Figure 7C). Consistently, the relative tumor growth ratio was reduced by 92.7% in G7, which was significantly lower than those in the other groups (Figure 7D). These results indicated that OMF‐guided delivery, AMF activation, and NIR‐II laser irradiation acted cooperatively to maximize the antitumor activity of NFSH nanozymes.

The in vivo thermal response was recorded by infrared thermal imaging during AMF treatment. As shown in Figures 7E,F, only a slight temperature increase was detected in G2, and the tumor‐site temperature remained below 40°C after 10 min of AMF treatment. In contrast, rapid temperature elevation was observed in the NFSH‐treated groups under AMF stimulation. The tumor temperature in G6 and G7 increased to approximately 46°C–47°C within 5–10 min. This controlled magnetothermal response was mainly ascribed to OMF‐enhanced tumor accumulation of NFSH and its magnetic energy‐conversion capacity. The temperature increase was sufficient to damage tumor cells and accelerate catalytic reactions, while no apparent body‐weight loss was observed in any group during treatment (Figure S24). These findings suggested that NFSH‐mediated magnetothermal activation could be achieved within a therapeutically effective and systemically tolerable range.

Histological and immunofluorescence analyses were performed to clarify the antitumor mechanism in tumor tissues. H&E staining showed dense tumor cell proliferation in G1 and G2, whereas obvious tissue destruction, nuclear shrinkage, and necrotic features were observed after NFSH‐based treatments (Figure 7G). The most severe tumor damage was detected in G7, which was consistent with the bioluminescence imaging results. TUNEL staining further confirmed that cell death was significantly increased after combined treatment. Quantitative analysis showed that the mean fluorescence intensity of TUNEL staining was highest in G7, reaching approximately 65%, while only weak TUNEL signals were observed in G1 and G2 (Figure 7H). These results demonstrated that the full combination of NFSH, OMF, AMF, and 1064 nm laser irradiation induced extensive tumor cell death in orthotopic GBM tissues. The tumor hypoxia status was then evaluated by HIF‐1α staining. Strong HIF‐1α expression was observed in the control groups, reflecting the hypoxic microenvironment of orthotopic GBM (Figure 7G). In contrast, HIF‐1α staining was weakened after NFSH‐mediated treatments, especially in G6 and G7. This decrease was attributed to the catalase‐like activity of NFSH, which could promote oxygen generation from endogenous H_2_O_2_ and thereby relieve tumor hypoxia. Hypoxia mitigation was expected to improve NIR‐II photodynamic reactions and to support oxidative stress amplification in tumor tissues.

Autophagy‐related resistance was investigated by P62 staining. P62 expression was markedly increased in NFSH‐treated groups and was highest in G7 (Figure 7G,I). Since P62 accumulation is commonly associated with impaired autophagic degradation, these results suggested that NFSH‐based therapy blocked autophagic flux in tumor tissues. This effect was particularly pronounced when AMF and 1064 nm laser irradiation were combined. The disrupted autophagic degradation was expected to weaken the repair capacity of GBM cells under oxidative and metabolic stress. Thus, the increased P62 signal supported the proposed role of NFSH in overcoming autophagy‐mediated therapeutic resistance. Ferroptosis‐related changes were further examined by GPX4 staining. Strong GPX4 expression was retained in G1 and G2, indicating an active antioxidant defense state in untreated tumors. In contrast, GPX4 expression was markedly decreased after NFSH‐based treatments and was most strongly suppressed in G7 (Figure 7G; Figure S25). Quantitative analysis showed that GPX4 fluorescence intensity was reduced from more than 70% in the control groups to a much lower level in G6 and G7. Since GPX4 is a key regulator that protects cells from lipid peroxidation, its downregulation indicated that ferroptosis defense was impaired. These results suggested that NFSH‐mediated cascade catalysis promoted ferroptosis by weakening GPX4‐dependent antioxidant protection. The NADH content in tumor tissues was also measured to evaluate metabolic disruption after treatment. Compared with the control groups, NADH levels were reduced by 7.8% in G3, 43.7% in G4, 52.4% in G5, 66.4% in G6, and 80.2% in G7 (Figure 7J). The sharp decrease in NADH in G7 indicated severe disturbance of intracellular redox homeostasis and energy metabolism. This NADH depletion could reduce ATP production, impair lysosomal function, and aggravate autophagic degradation blockade. Together with GPX4 downregulation, NADH depletion further supported the conclusion that NFSH + OMF + AMF + 1064 nm laser therapy amplified ferroptosis while suppressing autophagy‐associated repair pathways.

The survival outcomes further confirmed the therapeutic advantage of the combined treatment. As shown in Figure 7K, mice in G1 and G2 died rapidly because of uncontrolled tumor progression. NFSH‐based treatments prolonged survival to different degrees, and the greatest survival benefit was observed in G7. The survival curve of G7 remained higher than those of all other groups during the observation period, indicating that the coordinated action of OMF‐guided delivery, AMF activation, and 1064 nm laser irradiation translated into a meaningful therapeutic benefit in orthotopic GBM‐bearing mice. The biosafety of NFSH nanozymes was then systematically evaluated. The hemolysis assay showed that the hemolysis ratio remained below 0.3% even when the NFSH concentration was increased to 5 mg/mL, indicating favorable hemocompatibility (Figure S26A). After treatment, blood routine and biochemical analyses were performed. Hematological indices, including white blood cells (WBC), platelets (PLT), lymphocytes, hemoglobin (HGB), and granulocytes, showed no obvious abnormalities among the groups (Figure S26B–G). Liver and kidney function markers, including albumin (ALB), alkaline phosphatase (ALP), aspartate aminotransferase (AST), creatinine (CREA), uric acid (UA), and blood urea nitrogen, also remained within comparable ranges (Figure S26H–L). In addition, H&E staining of the heart, liver, spleen, lung, and kidney showed no apparent pathological damage, inflammatory infiltration, or tissue necrosis after different treatments (Figure S27).

### 2.8 In Vivo Mechanistic Analysis of NFSH‐Mediated Antitumor Therapy

**FIGURE 8:**
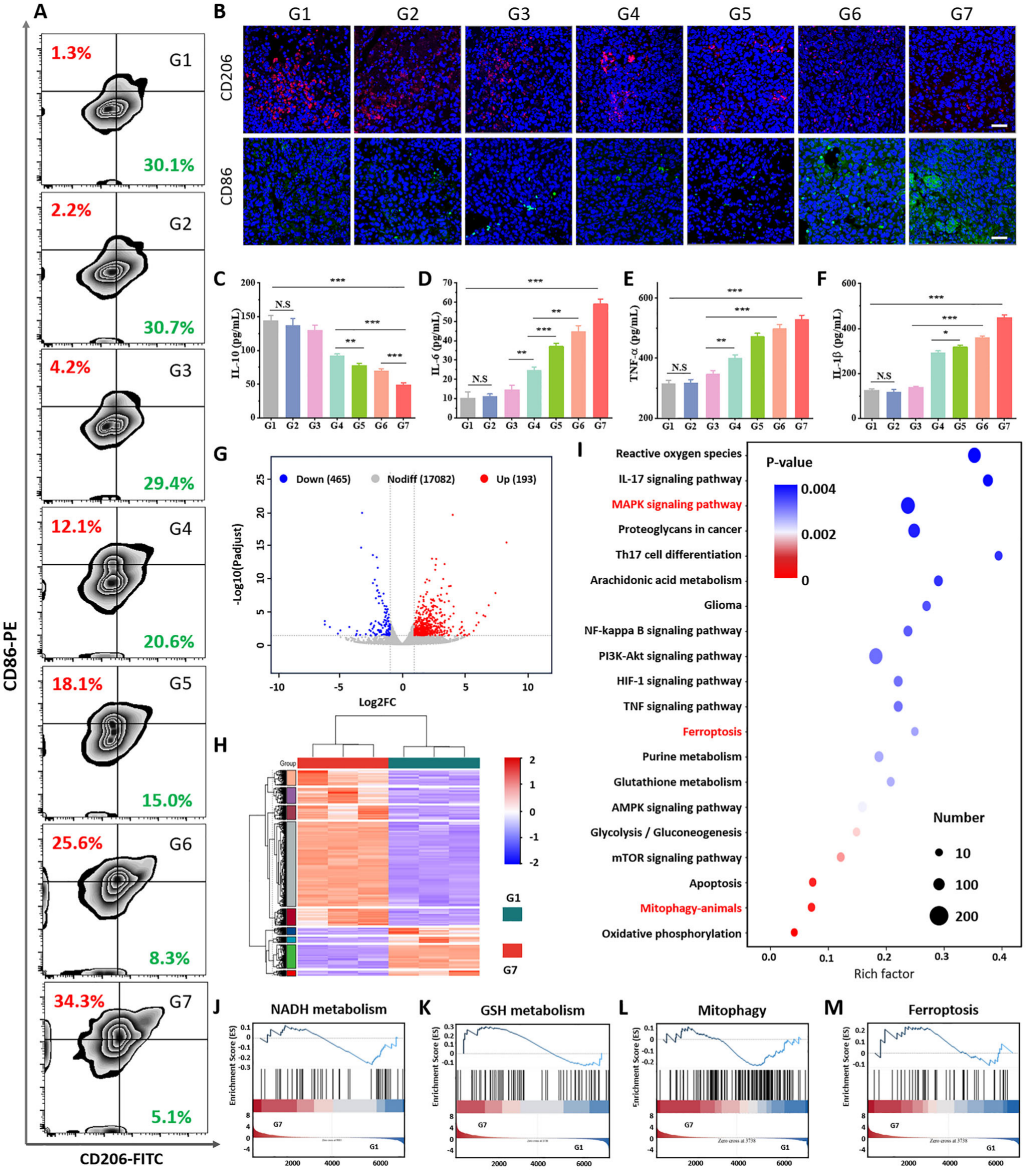
In vivo analysis of macrophage polarization, inflammatory factors, and transcriptomic changes after NFSH treatment. (A) Flow cytometry analysis of M1‐ and M2‐type macrophages in tumor tissues. (B) Immunofluorescence staining of CD206 and CD86 in tumor sections; scale bar: 100 µm. Tumor levels of (C) IL‐10, (D) IL‐6, (E) TNF‐α, and (F) IL‐1β after different treatments. (G) Volcano plot and (H) heatmap of differentially expressed genes (DEGs) between the NFSH + OMF + AMF + 1064 nm laser group (G7) and PBS group (G1). (I) KEGG enrichment analysis of DEGs between G7 and G1. GSEA of (J) NADH metabolism, (K) GSH metabolism, (L) mitophagy, and (M) ferroptosis in the G7 versus G1 comparison. Treatment groups were G1: PBS, G2: AMF + 1064 nm laser, G3: NFSH, G4: NFSH + OMF, G5: NFSH + OMF + 1064 nm laser, G6: NFSH + OMF + AMF, and G7: NFSH + OMF + AMF + 1064 nm laser. Unless otherwise indicated, the 1064 nm laser was applied at 0.33 W/cm^2^ for 10 min, AMF was applied at 495 kHz and 1.27 × 10^3^ A/m for 10 min, and OMF was applied using a cylindrical Nd‐Fe‐B magnet with a diameter of 2 cm, thickness of 1 cm, and magnetic field strength of 40 mT for 5 h. Data are shown as mean ± SD (*n* = 3). *p < 0.05, **p < 0.01, ***p < 0.001. N.S., not significant.

To validate the regulatory effect of NFSH‐mediated therapy on the TIME, tumor tissues were collected 10 days after treatment for macrophage phenotyping, cytokine analysis, and transcriptomic profiling. Tumor‐associated macrophage (TAM) polarization was examined by flow cytometry. As shown in Figure 8A, CD86^+^ M1‐like TAMs were gradually increased from G1 to G7, whereas CD206^+^ M2‐like TAMs were progressively reduced. Quantitative analysis further confirmed this trend (Figure S28). The proportion of CD86^+^ TAMs increased from 1.3% in G1 to 34.3% in G7, while the proportion of CD206^+^ TAMs decreased from 30.1% in G1 to 5.1% in G7. Compared with G1, the M2‐like TAM population in G7 was reduced by approximately 25 percentage points. Immunofluorescence staining of tumor sections showed consistent results (Figure 8B). Strong CD206 signals were observed in G1 and G2, whereas CD86 signals became increasingly prominent in the NFSH‐based combination groups, especially in G7. These results demonstrated that NFSH with OMF, AMF, and NIR‐II laser activation effectively promoted M2‐to‐M1 macrophage repolarization in orthotopic GBM tissues. Inflammatory cytokines in tumor tissues were then measured to evaluate changes in the GBM immune microenvironment. As displayed in Figure 8C, IL‐10, an immunosuppressive cytokine associated with tumor‐supportive inflammation, remained high in G1, G2, and G3. In contrast, IL‐10 was gradually decreased in the NFSH‐based combination groups and reached the lowest level in G7. Compared with G1, the IL‐10 level in G7 was reduced from approximately 140 pg/mL to nearly 50 pg/mL. This result indicated that the immunosuppressive component of the TIME was weakened after full magneto‐NIR‐II activation. By contrast, the proinflammatory cytokines IL‐6, TNF‐α, and IL‐1β were markedly increased after NFSH‐mediated therapy (Figure 8D–F). The highest levels of IL‐6, TNF‐α, and IL‐1β were detected in G7, reaching approximately 60, 520, and 450 pg/mL, respectively. These cytokine changes indicated that NFSH + OMF + AMF + 1064 nm laser treatment converted the GBM microenvironment from an immunosuppressive state toward an immune‐activated state.

RNA sequencing was performed to further clarify the molecular mechanisms underlying the NFSH‐mediated antitumor response. Gene expression profiles were compared between the PBS group (G1) and the NFSH + OMF + AMF + 1064 nm laser group (G7). The volcano plot showed that 658 differentially expressed genes (DEGs) were identified, including 193 upregulated genes and 465 downregulated genes in G7 compared with G1 (Figure 8G). The heatmap of DEGs showed a clear separation between G1 and G7, indicating that the full combination treatment induced a distinct transcriptional remodeling pattern in tumor tissues (Figure 8H). These results suggested that NFSH‐mediated therapy not only causes direct tumor cell damage but also broadly regulates immune signaling, redox metabolism, mitochondrial function, and cell‐death pathways. Kyoto Encyclopedia of Genes and Genomes (KEGG) pathway enrichment analysis was then conducted to identify biological processes associated with the DEGs. As shown in Figure 8I, the DEGs were enriched in pathways related to ROS, interleukin‐17 (IL‐17) signaling, mitogen‐activated protein kinase signaling, proteoglycans in cancer, T helper 17 (Th17) cell differentiation, arachidonic acid metabolism, glioma, nuclear factor kappa‐light‐chain‐enhancer of activated B cells (NF‐κB) signaling, phosphoinositide 3‐kinase–protein kinase B (PI3K–Akt) signaling, hypoxia‐inducible factor 1 (HIF‐1) signaling, tumor necrosis factor (TNF) signaling, ferroptosis, purine metabolism, glutathione metabolism, AMP‐activated protein kinase (AMPK) signaling, glycolysis/gluconeogenesis, mammalian target of rapamycin (mTOR) signaling, apoptosis, mitophagy, and oxidative phosphorylation. These enriched pathways closely matched the proposed therapeutic mechanism of NFSH. In particular, enrichment of ROS, glutathione metabolism, and ferroptosis pathways supported the role of cascade nanozyme catalysis in redox imbalance and ferroptotic damage. Enrichment of HIF‐1 signaling was consistent with hypoxia modulation, whereas changes in mitophagy, mTOR signaling, oxidative phosphorylation, and glycolysis/gluconeogenesis indicated that mitochondrial quality control and energy metabolism were strongly disturbed.

Cellular component GO enrichment analysis further revealed that the DEGs were mainly associated with organelle structures involved in metabolism, redox regulation, and autophagy. Enriched components included mitochondrion, mitochondrial inner membrane, mitochondrial matrix, mitochondrial envelope, peroxisome, peroxisomal membrane, lysosome, lysosome membrane, autophagosome, endocytic vesicle, exocytic vesicle, endoplasmic reticulum, and ATPase complex (Figure S29). These results suggested that NFSH‐mediated therapy affected mitochondria‐, lysosome‐, peroxisome‐, and autophagosome‐related structures. This organelle‐level remodeling was consistent with the proposed mechanism in which AMF‐promoted H_2_S release, NADH depletion, lysosomal dysfunction, and mitophagy interference jointly aggravated ferroptosis and weakened autophagic repair.

The expression profiles of representative genes further supported this mechanism. As shown in Figure S30, ferroptosis‐promoting genes, including Alox5, ACSL4, and ALOX15, were upregulated in G7, whereas ferroptosis‐suppressive and antioxidant defense genes, including GPX4, NRF2, and SLC7A11, were downregulated [66, 67, 68]. This expression pattern indicated that lipid peroxidation pathways were activated and the GPX4/SLC7A11/NRF2‐dependent ferroptosis‐defense system was weakened. Meanwhile, mitochondrial function‐ and mitophagy‐related genes, including Atg5, PRKN, Tfam, Tmem70, and Kcnq2, were reduced after treatment, while PINK1‐related signaling was increased [69, 70, 71, 72]. These changes suggested that mitochondrial stress was induced, but downstream mitophagic clearance and mitochondrial maintenance were impaired [73]. Therefore, NFSH‐mediated therapy appeared to uncouple mitochondrial damage from effective mitophagic repair, thereby increasing the vulnerability of GBM cells to ferroptosis.

Gene set enrichment analysis (GSEA) was further used to validate the major biological processes affected by NFSH treatment. NADH metabolism was markedly altered in G7 compared with G1 (Figure 8J), which agreed with the reduced NADH content detected in tumor tissues. This finding indicated that intracellular redox balance and mitochondrial energy metabolism were disrupted after treatment. GSH metabolism was also significantly changed (Figure 8K), supporting the involvement of glutathione oxidase‐like activity and antioxidant depletion in NFSH‐mediated therapy. In addition, mitophagy‐related gene sets were strongly affected (Figure 8L), which was consistent with P62 accumulation and mitophagy impairment observed in tumor tissues. Ferroptosis‐related gene sets were also enriched in the G7 versus G1 comparison (Figure 8M), further confirming that the combined treatment amplified ferroptotic tumor cell death.

Additional GSEA results provided further evidence for the upstream regulatory events involved in this therapeutic process. H_2_S metabolism was significantly altered in G7 compared with G1 (Figure S31), supporting the role of AMF‐triggered NFSH activation in regulating H_2_S‐related metabolic processes within the acidic tumor microenvironment. JAK/STAT3 signaling was also activated after treatment (Figure S32), indicating that cytokine‐related inflammatory signaling was involved in the immune response induced by NFSH‐mediated therapy. Moreover, macrophage M1 versus M2 polarization‐related gene sets were enriched in G7 (Figure S33), which was consistent with the flow cytometry and immunofluorescence results. These findings demonstrated that the combined treatment reshaped the TIME by reducing immunosuppressive TAM features and promoting an M1‐like, tumor‐suppressive macrophage phenotype [74, 75, 76]. This integrated mechanism explained the strong tumor suppression and survival benefit observed in the NFSH + OMF + AMF + 1064 nm laser group.

## 3 Conclusion

In summary, NFSH nanozymes were developed as a magneto‐NIR‐II‐programmed theranostic platform to address two major barriers in GBM therapy: inefficient BBB traversal and autophagy‐mediated therapeutic resistance. Through Nd^3+^ doping and HA modification, NFSH integrated OMF‐guided trans‐BBB delivery, CD44‐mediated tumor targeting, NIR‐II fluorescence imaging, T_2_‐weighted MRI, and AMF/NIR‐II‐activated cascade catalysis within one system. Under AMF and 1064 nm laser stimulation, NFSH amplified ROS generation, depleted GSH, relieved hypoxia, disrupted NADH metabolism, promoted H_2_S release, impaired lysosomal autophagic degradation, and suppressed mitophagy‐associated repair, thereby sensitizing GBM cells to ferroptosis. In orthotopic GBM‐bearing mice, this coordinated treatment markedly inhibited tumor progression, prolonged survival, and reshaped the tumor immune microenvironment by reducing immunosuppressive IL‐10, enhancing proinflammatory cytokine production, and driving M2‐to‐M1 macrophage repolarization, while maintaining favorable short‐term biosafety. Compared with conventional nanozyme or single‐field activation strategies, this work established a cascade design principle that couples magnetic navigation, NIR‐II activation, multienzyme catalysis, metabolic exhaustion, autophagy blockade, ferroptosis amplification, and immune remodeling.

## 4 Experimental Section

### 4.1 Materials

Iron(III) acetylacetonate [Fe(acac)_3_], iron(II) acetylacetonate [Fe(acac)_2_], 1‐hexadecanediol, and oleylamine (OM) were purchased from Shanghai Titan Technology Co., Ltd. (Shanghai, China). Neodymium(III) 2,4‐pentanedionate [Nd(acac)_3_], 3,3′,5,5′‐tetramethylbenzidine (TMB), glutathione (GSH), and reduced nicotinamide adenine dinucleotide (NADH) were obtained from Shanghai Aladdin Bio‐Chem Technology Co., Ltd. (Shanghai, China). 5,5′‐Dithiobis(2‐nitrobenzoic acid) (DTNB) and methylene blue hydrate (MB) were supplied by Shanghai Macklin Biochemical Technology Co., Ltd. (Shanghai, China). Chloroform (CHCl_3_), sulfur (S), hydrogen peroxide (H_2_O_2_), dimethyl sulfoxide (DMSO), and anhydrous ethanol were obtained from Xilong Scientific Co., Ltd. (Guangzhou, China). Hyaluronic acid (HA, *M*_w_ = 10 000) was provided by Bloomage Biotechnology Co., Ltd. (Jinan, China). Thiazolyl blue tetrazolium bromide (MTT), a ferrous ion assay kit (BC5415), penicillin‐streptomycin, a Calcein‐AM/PI double‐staining kit (CA1630), and a JC‐1 mitochondrial membrane potential kit (CA1310) were purchased from Solarbio (Beijing, China). 2‐(4‐Amidinophenyl)‐6‐indolecarbamidine dihydrochloride (DAPI), a monodansylcadaverine (MDC) autophagy staining assay kit (C30185), an ATP assay kit (S0026), a ROS assay kit (S0033S), a lipid peroxidation malondialdehyde (MDA) assay kit (S0131S), a bicinchoninic acid (BCA) protein assay kit (P0012), Lyso‐Tracker (C1046), Mito‐Tracker (C1048), and HRP‐labeled goat anti‐mouse IgG(H+L) (A0216) were purchased from Beyotime (Shanghai, China). The H_2_S probe (WPS‐5, MX5302) was obtained from Maokang Biotechnology Co., Ltd. (Shanghai, China). Rabbit anti‐CD206/FITC antibody (bs4727R), rabbit anti‐F4/80/Cy5.5 antibody, rabbit anti‐CD86/PE antibody (bs1035R), rabbit anti‐GPX4 antibody (bs3884R), rabbit anti‐LC3 antibody (bs24359R), and rabbit anti‐p62 antibody (bsm51287M) were purchased from Bioss (Beijing, China). Mouse interleukin (IL)‐6, IL‐10, IL‐12, and tumor necrosing factor α (TNF‐α) enzyme‐linked immunosorbent assay (ELISA) kits were obtained from Neobioscience Technology Co., Ltd.

### 4.2 Characterization

The morphology of the nanozymes was characterized using transmission electron microscopy (TEM; FEI, USA) and scanning electron microscopy (SEM; Hitachi, Japan). Crystal structures were analyzed by XRD (Rigaku SmartLab). Elemental composition and surface chemical states were characterized using XPS (Kratos, Japan), while functional groups were analyzed by Fourier transform infrared spectroscopy (FTIR; PerkinElmer, USA). Hydrodynamic size and zeta potential were measured using a Nano‐ZSE analyzer (Malvern, UK). Magnetic hysteresis loops were recorded using a vibrating sample magnetometer (Lake Shore 7404, USA) at 300 K over a magnetic field range of −20000–20000 Oe. NIR‐II fluorescence spectra were collected using a near‐infrared fluorescence spectrophotometer equipped with an 808 nm laser (Horiba, France). UV–vis–NIR absorption spectra were measured using a UV‐2600i spectrophotometer (Shimadzu, Japan). In vivo NIR‐II fluorescence and bioluminescence imaging were performed using a full‐spectrum biological imaging system (Biolight, China). T_2_‐weighted MRI was conducted using a clinical 3 T MRI scanner (Siemens, Germany).

### 4.3 Preparation of NFSH Nanozymes

Nd_0.02_Fe_2.98_S_4_ nanoparticles were synthesized using a modified thermal decomposition method. Briefly, Fe(acac)_2_ (0.127 g), Fe(acac)_3_ (0.353 g), and Nd(acac)_3_ (0.033 g) were added to a three‐neck flask containing OM (40 mL) and 1‐hexadecanediol (0.388 g). The mixture was heated to 120°C under Ar flow for 30 min to remove residual H_2_O and O_2_. Subsequently, the temperature was increased to 220°C until complete dissolution was achieved. Separately, sulfur powder (0.222 g) was dispersed in OM (10 mL) and heated to 70°C to form a transparent sulfur precursor solution. The sulfur solution was rapidly injected into the reaction system, and the mixture was heated to 260°C under an Ar atmosphere for 1 h. After cooling to room temperature, the products were washed three times with CHCl_3_/ethanol (3:1, v/v) and collected by centrifugation. The precipitates were redispersed in CHCl_3_ for storage. Fe_3_S_4_ nanoparticles were synthesized using the same procedure without Nd doping.

To prepare hydrophilic nanozymes, Nd_0.02_Fe_2.98_S_4_ nanoparticles (20 mg) were dispersed in CHCl_3_ (4 mL), while HA (50 mg) was dissolved in ultrapure water (10 mL). Under ultrasonication, the nanoparticle dispersion was slowly injected into the HA aqueous solution. The resulting emulsion was shaken at 37°C for 24 h and then centrifuged at 11 000 rpm for 20 min. The precipitates were washed three times with ultrapure water and freeze‐dried for 48 h to obtain NFSH nanozymes. For colloidal stability evaluation, NFSH nanozymes were dispersed in ultrapure water, PBS, and DMEM containing 10% FBS at a final concentration of 200 µg/mL. Hydrodynamic size changes were recorded over 7 days, and each measurement was repeated three times.

### 4.4 Magnetothermal Performance of NFSH Nanozymes

The magnetothermal performance of NFSH nanozymes was evaluated using a magnetic hyperthermia system (SPG800K2‐10B, China) equipped with a water‐cooled copper coil (70 mm inner diameter, 6 turns). The AMF frequency and intensity were set at 495 kHz and 1.27 × 10^3^ A/m, respectively. NFSH aqueous dispersions at concentrations of 0.125, 0.25, 0.5, 1, and 2 mg/mL were exposed to AMF for 10 min. Temperature changes were recorded using an infrared thermal imaging camera (Fluke, USA). Thermal cycling stability was evaluated over five consecutive heating/cooling cycles using a nanozyme concentration of 1 mg/mL.

The specific absorption rate (SAR) was calculated according to Equation (1):

(1) $$\mathrm{SAR}=\left(\Delta T/\Delta t\right)\times C/M_{Fe}$$

where *C* denotes the specific heat capacity of water (4.185 J/g °C), *M*_Fe_ represents the Fe mass (*g*), and Δ*T*/Δ*t* indicates the maximum heating rate (°C/s).

### 4.5 Singlet Oxygen Generation

The generation of singlet oxygen (^1^O_2_) by NFSH nanozymes under NIR‐II irradiation was evaluated using 1,3‐diphenylisobenzofuran (DPBF) as a probe. NFSH (200 µg/mL) and DPBF (25 µg/mL) were dispersed in buffer solution (pH 4.5) and irradiated with a 1064 nm laser at different power densities for 10 min. The supernatants were collected by centrifugation, and absorption spectra were recorded. The effects of H_2_O_2_ (0.1 mm) and AMF stimulation on ^1^O_2_ production were also investigated. Furthermore, electron spin resonance (ESR; Bruker ESR5000, Germany) was used to verify ^1^O_2_ generation using TEMPO as a trapping agent under different stimulation conditions, including 1064 nm laser irradiation (0.33 W/cm^2^), AMF exposure (495 kHz, 1.27 × 10^3^ A/m), and H_2_O_2_ addition (0.1 mm).

### 4.6 Multienzyme‐Like Activities of NFSH Nanozymes

The CAT‐like activity of NFSH nanozymes was evaluated using a dissolved oxygen meter (JPBJ‐608, China). NFSH (200 µg/mL) was dispersed in buffer solutions with pH values of 4.5, 5.5, 6.5, and 7.4. Subsequently, H_2_O_2_ (10 mm) was added, and dissolved oxygen concentrations were recorded every 30 s. The effects of H_2_O_2_ concentration and temperature on CAT‐like activity were also evaluated. Enzyme kinetics were analyzed in the presence or absence of AMF according to Equations (2) and (3):

(2) $$V=\left(V_{\max}\times S\right)/\left(K_{m}+S\right)$$

(3) $$V=\Delta A/\left(\Delta t\times \varepsilon \times l\right)$$

where *V* exhibits the reaction rate (mm/s), *V*_max_ displays the maximum reaction rate (mm/s), *S* demonstrates substrate concentration (mm), *K*_m_ represents the Michaelis–Menten constant, Δ*A* shows absorbance change, Δ*t* indicates reaction time (s), *ε* denotes the molar extinction coefficient, and l represents the optical path length (1 cm).

The POD‐like activity of NFSH nanozymes was evaluated using the oxidation of TMB in the presence of H_2_O_2_. NFSH (200 µg/mL) was dispersed in buffer solutions with different pH values and incubated with TMB (1.5 mg/mL) and H_2_O_2_ (0.1 mm). Absorption spectra were subsequently recorded. The effects of nanozyme concentration and AMF stimulation on POD‐like activity were further evaluated. The GSHOx‐like activity was evaluated by monitoring GSH consumption using DTNB. NFSH (200 µg/mL) was incubated with GSH (1 mm) in buffer solutions with different pH values for 10 min. After DTNB addition, absorbance at 422 nm was recorded. The effects of concentration and AMF stimulation on GSH depletion were also investigated. The NOX‐like activity of NFSH nanozymes was evaluated by monitoring NADH oxidation using UV–vis spectroscopy. NFSH (200 µg/mL) and NADH (50 µg/mL) were incubated in buffer solutions with different pH values for 10 min. Absorption changes were recorded before and after AMF exposure (495 kHz, 1.27 × 10^3^ A/m). The generation of H_2_O_2_ induced by NOX‐like activity was further confirmed using TMB oxidation assays at different temperatures (37°C, 42°C, and 47°C) and pH values. Systems without NFSH served as controls.

### 4.7 Fe Ion and H_2_S Release

Fe ion release from NFSH nanozymes was evaluated using dialysis experiments. NFSH (5 mg) was dispersed in 10 mL of buffer solution with different pH values and transferred into dialysis bags (MWCO: 5 kDa). At predetermined time points, dialysates were collected for ICP‐MS analysis and replenished with fresh buffer. The effects of temperature (37°C, 42°C, and 47°C) on Fe release were also investigated.

H_2_S release was evaluated using an MB fading assay. NFSH nanozymes (20 mg) were dispersed in 20 mL of MB‐containing buffer solution (0.2%, w/v) with different pH values. After oscillation for 120 min, supernatants were collected for UV–vis analysis. Concentration‐dependent, AMF‐enhanced, and temperature‐dependent H_2_S release profiles were further investigated.

### 4.8 Cell Culture

L929, GL261, bEnd.3, RAW264.7, and GL261‐Luc cells were purchased from Hunan Fenghui Biotechnology Co., Ltd. (Changsha, China). Cells were cultured in DMEM supplemented with 10% FBS and 1% penicillin‐streptomycin at 37°C in a humidified atmosphere containing 5% CO_2_.

### 4.9 Cellular Uptake

GL261 cells (5 × 10^4^ cells/well) were seeded in confocal dishes and incubated for 12 h. After washing with PBS, the medium was replaced with fresh medium containing FITC‐labeled NFSH nanozymes (200 µg/mL). To evaluate the effect of a static magnetic field on cellular uptake, a cylindrical Nd‐Fe‐B magnet (40 mT) was placed beneath the culture dish. After incubation for different time intervals, the cells were fixed, stained with DAPI, and imaged using confocal laser scanning microscopy (CLSM; Stellaris Leica, Switzerland).

### 4.10 Penetration Efficiency in Multicellular Tumor Spheroids

GL261 multicellular tumor spheroids (MTSs) were established according to previously reported protocols. Briefly, GL261 cells (2 × 10^3^ cells/well) were seeded in 96‐well plates precoated with 1.5% agarose and cultured for 5–7 days, with half of the medium replaced every 48 h. When the spheroid diameter reached approximately 300 µm, an Nd‐Fe‐B magnet was placed beneath the culture plate, and FITC‐labeled NFSH nanozymes (200 µg/mL) were added. After incubation for 8, 12, 24, or 36 h, the spheroids were transferred to confocal dishes and analyzed by CLSM. Penetration efficiency was calculated according to Equation (4):

(4) Penetrationefficiency=r/R×100%

where *R* is the maximum cross‐sectional radius of the MTS, and *r* corresponds to the radius of the fluorescence‐positive region at a penetration depth of 70 µm.

### 4.11 Cytotoxicity of NFSH Nanozymes

L929, bEnd.3, and GL261 cells (5 × 10^3^ cells/well) were seeded in 96‐well plates and incubated at 37°C for 12 h. After the medium was removed, fresh medium containing different concentrations of NFSH nanozymes (0, 3.1, 6.3, 12.5, 25, 50, 100, 200, and 400 µg/mL) was added. The cells were incubated for another 24 h in a humidified 5% CO_2_ incubator. Subsequently, MTT solution (20 µL, 5 mg/mL) was added to each well, and the cells were incubated at 37°C for 4 h. After the medium was discarded, DMSO (200 µL) was added to dissolve the formazan crystals. Absorbance at 490 nm was measured using a microplate reader (Tecan Spark, Switzerland), and cell viability was calculated according to Equation (5):

(5) $$\mathrm{Cell}\;\mathrm{viability}\left(100\%\right)=\frac{{\mathrm{OD}}_{490sample}-{\mathrm{OD}}_{490blank}}{{\mathrm{OD}}_{490control}-{\mathrm{OD}}_{490blank}}\times 100\%$$

where OD_490sample_ represents the absorbance of cells treated with NFSH nanozymes at 490 nm, OD_490blank_ exhibits the absorbance of blank wells without cells, and OD_490control_ indicates the absorbance of untreated control cells.

### 4.12 In Vitro Antitumor Effect

The in vitro antitumor effect of NFSH nanozymes was evaluated using the MTT assay. GL261 cells (2 × 10^4^ cells/dish) were seeded in 30 mm cell culture dishes and incubated for 24 h. The medium was replaced with DMEM containing NFSH nanozymes (200 µg/mL), and the cells were incubated for another 6 h. The cells were then exposed to AMF (495 kHz, 1.27 × 10^3^ A/m, 10 min), 1064 nm laser irradiation (0.33 W/cm^2^, 10 min), or combined AMF/1064 nm laser stimulation. After incubation for 24 h, cell viability was evaluated using the MTT assay as described above.

Live/dead staining was used to further assess the antitumor effect of NFSH nanozymes. GL261 cells (1 × 10^5^ cells/dish) were seeded in 30 mm cell culture dishes and cultured for 12 h. The cells were treated with PBS, 1064 nm laser + AMF, NFSH, NFSH + 1064 nm laser, NFSH + AMF, or NFSH + 1064 nm laser + AMF. The NFSH concentration was 200 µg/mL. AMF stimulation (495 kHz, 1.27 × 10^3^ A/m) and 1064 nm laser irradiation (0.33 W/cm^2^) were applied for 10 min. After another 4 h of incubation, the cells were stained with Calcein‐AM/PI and observed using an inverted microscope (Leica, Switzerland).

The antitumor effect of NFSH nanozymes against MTSs was evaluated using the same treatment groups. The NFSH concentration was 200 µg/mL, and AMF stimulation (495 kHz, 1.27 × 10^3 ^A/m) and 1064 nm laser irradiation (0.33 W/cm^2^) were applied for 10 min. Morphological changes in the spheroids were recorded every 2 days using an inverted microscope (Leica, Switzerland).

### 4.13 Oxygen Production Assessment

GL261 cells were seeded in confocal dishes and incubated for 12 h. Hypoxia was then induced using a sealed vessel containing a hypoxia pouch for 1 h. The cells were loaded with the oxygen‐sensitive probe RDPP for 20 min and then incubated with NFSH nanozymes (200 µg/mL) for 6 h. The cells were treated with AMF (495 kHz, 1.27 × 10^3^ A/m, 10 min), 1064 nm laser irradiation (0.33 W/cm^2^, 10 min), or combined AMF/1064 nm laser stimulation. After washing with PBS, CLSM imaging was performed under 488 nm excitation. Intracellular oxygen levels were evaluated according to RDPP red fluorescence intensity, where stronger fluorescence indicated lower oxygen concentration.

### 4.14 Intracellular ROS Detection

GL261 cells (1 × 10^5^ cells/dish) were seeded in confocal dishes and incubated for 12 h. After coincubation with NFSH nanozymes (200 µg/mL) for 6 h, the cells were treated with AMF (495 kHz, 1.27 × 10^3^ A/m, 10 min), 1064 nm laser irradiation (0.33 W/cm^2^, 10 min), or combined AMF/1064 nm laser stimulation. After washing with PBS, intracellular ROS were stained using a ROS assay kit according to the manufacturer's protocol and observed by CLSM.

### 4.15 In Vitro BBB Permeability Assessment

An in vitro BBB model was constructed using a Transwell culture system. bEnd.3 cells (1 × 10^5^ cells/well) were seeded in the upper chamber to form tight junctions. When the transepithelial/transendothelial electrical resistance (TEER) reached 200 Ω cm^2^, GL261 cells (1 × 10^5^ cells/well) were seeded in the lower chamber and incubated for 12 h. FITC‐labeled NFSH nanozymes (200 µg/mL) were then added to the upper chamber, and a cylindrical Nd‐Fe‐B magnet (40 mT) was placed beneath the lower chamber. At predetermined time points, GL261 cells were washed with PBS, stained with DAPI, and analyzed by CLSM.

### 4.16 NADH Level Detection

Intracellular NADH levels were quantified using a commercial assay kit. GL261 cells were treated according to the MTT treatment protocol described above. After an additional 24 h of incubation, the cells were lysed and centrifuged at 2000 rpm for 5 min. The supernatants were collected, and NADH levels were measured according to the manufacturer's instructions.

### 4.17 ATP Level Detection

Intracellular ATP levels in GL261 cells were measured using a commercial ATP assay kit. After the cells were treated according to the MTT treatment protocol, they were incubated for another 24 h and then lysed. The lysates were collected by centrifugation at 2000 rpm for 5 min, and ATP levels were quantified according to the kit instructions.

### 4.18 Mitochondrial Membrane Potential Assay

Mitochondrial membrane potential was evaluated using JC‐1 staining. GL261 cells were seeded in confocal dishes, incubated with NFSH nanozymes for 6 h, and then exposed to 1064 nm laser irradiation (0.33 W/cm^2^, 10 min), AMF stimulation (495 kHz, 1.27 × 10^3^ A/m, 10 min), or combined stimulation. After another 6 h of incubation, the cells were stained with JC‐1 dye and washed with PBS. Before CLSM imaging, the nuclei were counterstained with DAPI.

### 4.19 Intracellular H_2_S and Autophagy Level Detection

GL261 cells (5 × 10^4^ cells/dish) were seeded in confocal dishes and incubated for 12 h. The cells were treated with NFSH nanozymes (200 µg/mL) and exposed to different stimulation conditions. After another 6 h of incubation, the cells were washed with PBS and stained using WPS‐5 and MDC assay kits according to the manufacturer's instructions. Fluorescence signals were detected by CLSM.

### 4.20 Western Blot Analysis

The expression levels of autophagy‐related proteins were analyzed by western blotting. GL261 cells subjected to different treatments were lysed and collected. Total protein concentrations were quantified using a BCA assay kit. Proteins were separated by SDS‐PAGE and transferred onto PVDF membranes. The membranes were blocked with a rapid blocking solution and incubated with primary antibodies against GAPDH, p62, and LC3 for 12 h. After washing, the membranes were incubated with HRP‐labeled goat anti‐mouse IgG(H + L) secondary antibody. Protein bands were visualized using enhanced chemiluminescence and imaged with a gel documentation system (GE, USA).

### 4.21 Mitochondrial and Lysosomal Staining Analysis

GL261 cells were seeded in confocal dishes and treated with PBS, 1064 nm laser + AMF, NFSH, NFSH + 1064 nm laser, NFSH + AMF, NFSH + 1064 nm laser + AMF, or NaHS. The concentrations of NFSH and NaHS were 200 µg/mL and 126 µm, respectively. AMF stimulation (495 kHz, 1.27 × 10^3^ A/m) and 1064 nm laser irradiation (0.33 W/cm^2^) were applied for 10 min. After another 6 h of incubation, the cells were stained with Lyso‐Tracker and Mito‐Tracker for 30 min. Finally, the nuclei were stained with Hoechst for 15 min, and the cells were observed by CLSM.

### 4.22 Ferroptosis Assessment

GSH depletion induced by NFSH nanozymes was first quantified using a commercial GSH assay kit. GL261 cells were seeded in 30 mm dishes and cultured for 12 h. The cells were then treated with NFSH nanozymes (200 µg/mL) for 6 h and exposed to the corresponding stimulation conditions. The cells were collected and lysed, and GSH levels were measured according to the manufacturer's protocol. GSH consumption under different stimulation conditions was analyzed using the same method. GPX4 expression was evaluated by immunofluorescence staining. After different treatments, the cells were incubated with anti‐GPX4 antibody for 2 h. After washing with PBS, the cells were incubated with Alexa Fluor 488‐conjugated secondary antibody for 30 min and stained with DAPI. Images were acquired by CLSM. In addition, GL261 cells were lysed and centrifuged, and MDA levels were determined using an MDA assay kit.

### 4.23 Fe^2+^ Ion Level Detection

Intracellular Fe^2+^ levels were quantified using a commercial ferrous ion assay kit. GL261 cells were treated according to the MTT treatment protocol and incubated for another 24 h before lysis. The lysates were collected by centrifugation at 2000 rpm for 5 min, and Fe^2+^ levels were measured according to the manufacturer's instructions.

### 4.24 In Vitro Macrophage Polarization

RAW264.7 macrophages (5 × 10^4^ cells/dish) were seeded in 35 mm culture dishes and cultured for 24 h. Fresh medium containing IL‐4 (200 ng/mL) was then added, and the cells were cultured for another 48 h to induce M2‐like polarization. After washing, medium containing NFSH nanozymes (50 µg/mL) was added. The cells were then exposed to AMF (495 kHz, 1.27 × 10^3^ A/m, 10 min), 1064 nm laser irradiation (0.33 W/cm^2^, 10 min), or combined AMF/1064 nm laser stimulation. The cells were collected and stained with anti‐CD206/FITC and anti‐CD86/PE antibodies for 20 min. Macrophage phenotypes were analyzed by flow cytometry (Beckman, USA).

### 4.25 Animal Experiments

All animal experiments were performed in accordance with the guidelines of the Hainan University Institutional Animal Use and Care Committee (Approval No. HNUAUCC‐2024‐00275). Female C57BL/6J mice (5–6 weeks old, 17–21 g) were purchased from Sibeifu Biotechnology Co., Ltd. (Beijing, China).

### 4.26 Establishment of Orthotopic Glioblastoma Models

C57BL/6J mice were anesthetized by intraperitoneal injection of 1% pentobarbital sodium and fixed in a stereotaxic apparatus (Zhongshi Technology, Beijing, China). GL261‐Luc cells (5 × 10^5^ cells in 10 µL PBS) were injected into the right striatum at a rate of 2 µL/min. The injection coordinates were 2 mm lateral to the bregma, 1.5 mm anterior to the bregma, and 2.5 mm below the skull surface. *D*‐Luciferin potassium salt (150 mg/kg body weight) was intraperitoneally injected to monitor tumor growth by bioluminescence imaging.

### 4.27 In Vivo Antitumor Evaluation

Mice bearing orthotopic glioblastoma were randomly divided into seven groups (*n* = 6): G1, PBS; G2, AMF + 1064 nm laser; G3, NFSH; G4, NFSH + OMF; G5, NFSH + OMF + 1064 nm laser; G6, NFSH + OMF + AMF; and G7, NFSH + OMF + AMF + 1064 nm laser. NFSH nanozymes (15 mg/kg) were intravenously injected into mice in groups G3–G7. After injection, a circular Nd‐Fe‐B magnet (40 mT) was fixed above the brain region of mice in groups G4–G7 to enhance BBB permeability. After 5 h, mice in groups G2, G5, and G7 were irradiated with a 1064 nm laser (0.33 W/cm^2^) for 10 min, while mice in groups G2, G6, and G7 received AMF stimulation (495 kHz, 1.27 × 10^3^ A/m) for 10 min. Treatments were performed on day 0 and day 3. Tumor growth was monitored by bioluminescence imaging on days 0, 5, 10, and 14. Body weight was recorded every 2 days during treatment. After treatment, brain tissues from different groups were collected for hematoxylin and eosin (H&E) staining, TUNEL staining, and immunofluorescence staining of GPX4 and p62.

On day 10 of treatment, tumor tissues from different groups were collected and dissociated into single‐cell suspensions. The cells were incubated with fluorescently labeled antibodies, including rabbit anti‐CD206/FITC, rabbit anti‐F4/80/Cy5.5, and rabbit anti‐CD86/PE antibodies, for 30 min. The proportion and phenotype of TAMs were analyzed by flow cytometry. Immunostaining of tumor tissues for CD86 and CD206 was performed to further evaluate macrophage infiltration and polarization. Serum samples were collected from mice in different treatment groups, and IL‐6, IL‐10, IL‐12, and TNF‐α levels were quantified using ELISA kits according to the manufacturer's protocols.

After 14 days, the mice were euthanized. Major organs, including the heart, liver, spleen, lungs, and kidneys, were harvested and fixed with 4% paraformaldehyde. The tissues were embedded in paraffin, sectioned, and stained with H&E. Blood samples were collected for routine blood tests and serum biochemical analyses.

### 4.28 Transcriptomic Analysis

Mice bearing orthotopic glioblastoma were randomly divided into two groups (*n* = 6): the PBS group (G1) and the NFSH + OMF + AMF + 1064 nm laser group (G7). Treatments were administered according to the protocol described above. On day 10 after treatment, the mice were euthanized, and tumor tissues were dissected and immediately flash‐frozen in liquid nitrogen. Transcriptomic analysis, including RNA extraction, reverse transcription, library preparation, and sequencing, was performed by Personal Biotechnology Co., Ltd. (Shanghai, China). Data processing and visualization were conducted using the Personal Genecloud online platform.

### 4.29 Statistical Analysis

All experiments were independently repeated at least three times per group. Data were presented as mean ± SD. Statistical analyses were performed using Student's *t*‐test for comparisons between two groups and one‐way ANOVA for comparisons among multiple groups. Statistical significance was defined as follows: N.S., not significant; *p < 0.05; **p < 0.01; and ***p < 0.001.

## Author Contributions

**Ruocan Liu**: conceptualizations, investigation, formal analysis, data curation, methodology, and Writing – original draft. **Yundi Wu**: conceptualizations, investigation, data curation, validation, project administration, and writing – review & editing. **Shuai Zhang**: data curation, methodology, visualization, and software. **Hongjuan Zhao**: formal analysis, investigation, and methodology. **Jiping Zheng**: resources, methodology, and validation. **Shiyang Shao**: formal analysis and data curation. **Jianqiang Chen**: methodology and validation. **Xilong Wu**: conceptualization, formal analysis, funding acquisition, supervision, project administration, resources, and writing – review & editing.

## Conflicts of Interest

The authors declare no conflicts of interest.

## Supporting information




**Supporting File**: advs76998‐sup‐0001‐SuppMat.docx.

## Data Availability

The data that support the findings of this study are available from the corresponding author upon reasonable request.
