# Supplementary material for: Magneto‐NIR‐II‐Programmed Cascade Nanozymes Unlocking Blood–Brain Barrier Translocation and Autophagic Resistance in Glioblastoma
Source: Adv Sci (Weinh). 2026 Aug 5;13(60):e76998. doi: 10.1002/advs.76998 (PMC13440201; doi:10.1002/advs.76998)
Supplement: Supplementary file 1 [file ADVS-13-e76998-s001.docx]

***Supporting Information***

**Magneto-NIR-II-Programmed Cascade Nanozymes Unlocking Blood-Brain Barrier Translocation and Autophagic Resistance in Glioblastoma**

**Ruocan Liu^a,b,d^†, Yundi Wu^a,b,c^†, Shuai Zhang^a,b^, Hongjuan Zhao^a,b^, Jiping Zheng^d^, Shiyang Shao^e^, Jianqiang Chen^c^, Xilong Wu^a,b*^**

^a^ State Key Laboratory of Digital Medical Engineering, School of Biomedical Engineering, School of Pharmaceutical Sciences, Hainan University, Haikou 570228, China

^b^ Key Laboratory of Tropical Biological Resources of Ministry of Education, Hainan Engineering Research Center for Drug Screening and Evaluation, Collaborative Innovation Center of One Health, Hainan University, Haikou 570228, China

^c^ Key Laboratory of Emergency and Trauma of Ministry of Education, Engineering Research Center for Hainan Biological Sample Resources of Major Diseases, The First Affiliated Hospital, Hainan Medical University, Haikou 571199, China

^d^ School of Life and Health Sciences, Collaborative Innovation Center of Life and Health, Hainan University, Haikou 570228, China

^e^ School of Materials Science and Engineering, Hainan University, Haikou 570228, China

†These authors contributed equally to this work.

***Corresponding author**

E-mail: xilongwu@foxmail.com (X. Wu);

**Figure S1.** Morphological characterization of Nd_0.02_Fe_2.98_S_4_ nanomaterials. A) Scanning electron microscopy (SEM) image of Nd_0.02_Fe_2.98_S_4_ nanomaterials. B) Particle size distribution of Nd_0.02_Fe_2.98_S_4_ nanomaterials measured from panel A (n = 200 particles).

**Figure S2.** Elemental contents of Nd_0.02_Fe_2.98_S_4_ nanomaterials determined by energy-dispersive X-ray spectroscopy (EDS) mapping.

**Figure S3.** SEM images of NFSH nanozymes.

**Figure S4.** XPS survey spectrum of NFSH nanozymes.

**Figure S5.** Colloidal stability of NFSH nanozymes. Hydrodynamic size changes of NFSH dispersed in different media over 7 d. Data are presented as means ± SD (n = 3).

**Figure S6.** Magnetic-field response of NFSH nanozymes. Photographic images showing the distribution of NFSH nanozymes in aqueous solution before and after exposure to a 40 mT magnetic field.

**Figure S7.** Specific absorption rate (SAR) values of NFSH measured under AMF treatment at different magnetic field intensities.

**Figure S8.** Valence-band XPS spectra of A) Fe_3_S_4_ and B) NFSH.

**Figure S9.** Detection of ^1^O_2_ generation by NFSH under different irradiation conditions. A) UV-vis absorption spectra of DPBF for detecting ^1^O_2_ generation in the presence of NFSH under 1064 nm laser irradiation at different power densities. B) UV-Vis absorption spectra of DPBF for detecting ^1^O_2_ generation in the presence of NFSH under different stimulation conditions. Unless otherwise indicated, NFSH was used at 200 μg/mL, H_2_O_2_ at 0.1 mM, the 1064 nm laser at 0.33 W/cm^2^ for 10 min, and AMF at 495 kHz and 1.27 × 10^3^ A/m for 10 min. Data are presented as means ± SD (n = 3).

**Figure S10.** O_2_ generation by NFSH in H_2_O_2_-containing solution. O_2_ levels in pH 7.4 aqueous solution containing A) different concentrations of NFSH with 10 mM H_2_O_2_ or B) NFSH at 200 μg/mL with different concentrations of H_2_O_2_.

**Figure S11.** POD-like catalytic activity of NFSH under different substrate conditions. UV-vis absorbance spectra of TMB aqueous solution at pH 4.5 containing A) different concentrations of NFSH with 0.1 mM H_2_O_2_ or B) NFSH at 200 μg/mL with different concentrations of H_2_O_2_.

**Figure S12.** GSHOx-like catalytic activity of NFSH nanozymes. A) pH-dependent and B) concentration-dependent GSH consumption by NFSH. C) Absorbance changes at 422 nm in mixed solutions of DTNB and GSH under different conditions. D) Michaelis-Menten curves of NFSH using GSH as the substrate. AMF was applied at 495 kHz and 1.27 × 10^3^ A/m for 10 min where indicated. Data are presented as means ± SD (n = 3).

**Figure S13.** Fe ion release from NFSH under different incubation conditions. Fe ion release from NFSH after incubation in buffer solutions with different temperatures and pH values for 120 min, as measured by ICP-MS. Data are presented as means ± SD (n = 3).

**Figure S14.** Concentration-dependent H_2_S release from NFSH. Absorption spectra of MB solutions used to detect H_2_S release from different concentrations of NFSH after 120 min of reaction; photographs show the corresponding color changes.

**Figure S15.** Temperature-dependent H_2_S release from NFSH. Absorption spectra of mixed solutions containing MB and NFSH at 200 μg/mL at pH 4.5 under different temperatures after 120 min of reaction.

**Figure S16.** In vitro therapeutic evaluation of NFSH in MTSs. Representative images of MTSs after different treatments for 5 d and the corresponding quantitative analysis of MTS volumes; scale bar: 100 μm; n = 3. Treatment groups were I: PBS, II: 1064 nm laser+AMF, III: NFSH, IV: NFSH+1064 nm laser, V: NFSH+AMF, and VI: NFSH+1064 nm laser+AMF. Unless otherwise indicated, NFSH was used at 200 μg/mL, the 1064 nm laser at 0.33 W/cm^2^ for 10 min, and AMF at 495 kHz and 1.27 × 10^3^ A/m for 10 min. Data are presented as means ± SD (n = 3). *p < 0.05, **p < 0.01, ***p < 0.001.

**Figure S17.** Quantitative analysis of P62 expression in GL261 cells after treatment with different formulations. Data are presented as means ± SD (n = 3). **p < 0.01, ***p < 0.001.

**Figure S18.** Quantitative fluorescence intensity analysis of Lyso-Tracker/Hoechst staining from CLSM images. Data are presented as means ± SD (n = 3). *p < 0.05, ***p < 0.001. N.S., not significant.

**Figure S19.** Quantitative fluorescence intensity analysis of Mito-Tracker/Hoechst staining from CLSM images. Data are presented as means ± SD (n = 3). **p < 0.01, ***p < 0.001. N.S., not significant.

**Figure S20.** Quantitative fluorescence intensity analysis of intracellular GPX4 expression from CLSM images. Data are presented as means ± SD (n = 3). **p < 0.01, ***p < 0.001. N.S., not significant.

**Figure S21.** Intracellular Fe^2+^ contents in GL261 cells after different treatments. Treatment groups were I: PBS, II: AMF+1064 nm laser, III: NFSH, IV: NFSH+1064 nm laser, V: NFSH+AMF, and VI: NFSH+1064 nm laser+AMF. Unless otherwise indicated, NFSH was used at 200 μg/mL, the 1064 nm laser at 0.33 W/cm^2^ for 10 min, and AMF at 495 kHz and 1.27 × 10^3^ A/m for 10 min. Data are presented as means ± SD (n = 3). **p < 0.01, ***p < 0.001. N.S., not significant.

**Figure S22.** Flow cytometry analysis of macrophage polarization. CD86⁺ cells were used to identify M1-type macrophages, and CD206^+^ cells were used to identify M2-type macrophages. Treatment groups were I: PBS, II: AMF+1064 nm laser, III: NFSH, IV: NFSH+1064 nm laser, V: NFSH+AMF, and VI: NFSH+1064 nm laser+AMF. Unless otherwise indicated, NFSH was used at 200 μg/mL, the 1064 nm laser at 0.33 W/cm^2^ for 10 min, and AMF at 495 kHz and 1.27 × 10^3^ A/m for 10 min.

**Figure S23.** Quantitative biodistribution in tumors and major organs (A) with or (B) without OMF treatment. T: tumor; H: heart; Li: liver; S: spleen; Lu: lung; K: kidney. Data are expressed as means ± SD (n = 5). *p < 0.05, **p < 0.01, ***p < 0.001.

**Figure S24.** Body weight changes of mice in different treatment groups during the treatment period. Data are expressed as means ± SD (n = 6).

**Figure S25.** Quantitative fluorescence intensity analysis of GPX4 staining in tumor sections from different treatment groups. Data are presented as means ± SD (n = 5). *p < 0.05, ***p < 0.001.

**Figure S26.** Hemocompatibility and blood biochemical analysis of NFSH. A) Hemolysis ratios of red blood cells incubated with different concentrations of NFSH nanozymes (n = 3). Blood biochemical analysis of mice in different treatment groups, including B) white blood cells (WBC), C) platelets (PLT), D) lymphocytes (Lymph), E) hemoglobin (HGB), F) granulocytes (Gran), G) albumin (ALB), H) alkaline phosphatase (ALP), I) aspartate aminotransferase (AST), J) creatinine (CREA), K) uric acid (UA), and L) blood urea nitrogen (BUN). Data are presented as means ± SD (n = 5).

**Figure S27.** Histological analysis of major organs. H&E staining images of major organs collected from mice in different treatment groups; scale bar: 100 μm.

**Figure S28.** Quantitative analysis of the proportions of A) M1-type macrophages and B) M2-type macrophages from the flow cytometry analysis shown in Figure 8E. Data are shown as mean ± SD (n = 3). **p < 0.01, ***p < 0.001. N.S., not significant.

**Figure S29.** Cellular component Gene Ontology (GO) enrichment analysis of DEGs in the NFSH+OMF+AMF+1064 nm laser group (G7) compared with the PBS group (G1).

**Figure S30.** Heatmap of gene expression profiles before and after treatment. Heatmap showing gene expression profiles in the PBS group (G1) and NFSH+OMF+AMF+1064 nm laser group (G7).

**Figure S31.** Gene set enrichment analysis of H_2_S metabolism in the G7 versus G1 comparison.

**Figure S32**. Gene set enrichment analysis of JAK/STAT3 signaling in the G7 versus G1 comparison.

**Figure S33.** Gene set enrichment analysis of macrophage polarization-related gene sets in the G7 versus G1 comparison.

**Table S1.** Elemental composition of NFSH nanozymes.

| Samples | Fe (μmol/L) | Nd (μmol/L) | S (μmol/L) |
| --- | --- | --- | --- |
| NFSH-1 | 124.23 ± 1.15 | 0.91 ± 0.09 | 167.46 ± 2.15 |
| NFSH-2 | 119.79 ± 2.09 | 0.78 ± 0.12 | 168.17 ± 1.97 |
| NFSH-3 | 123.65 ± 2.11 | 0.83 ± 0.10 | 165.91 ± 1.73 |

**Table S2.** Kinetic parameters for POD-like catalytic activity.

| Sample | V_max_ (μM/s) | K_m_ (mM) | k_cat_ (s^-1^) | k_cat_/K_m_ (s^-1^/M) |
| --- | --- | --- | --- | --- |
| Fe_3_S_4_ | 0.42 | 0.649 | 1.4🞨10^4^ | 2.99🞨10^7^ |
| HRP | 0.0031 | 10.35 | 3.00🞨10^3^ | 2.90🞨10^5^ |
| NFSH | 0.63 | 0.32 | 5.20🞨10^4^ | 1.63🞨10^8^ |
| NFSH+AMF | 0.72 | 0.19 | 6.29🞨10^4^ | 3.31🞨10^8^ |
